# Regulation of redox homeostasis by ATF4-MTHFD2 axis during white adipose tissue browning

**DOI:** 10.1016/j.redox.2025.103715

**Published:** 2025-06-09

**Authors:** Rehna Paula Ginting, Hoang-Anh Pham-Bui, Choijamts Munkhzul, Siti Aisyah Fuad, Ahyeon Son, Jong-Seok Moon, Jaeseok Han, Mihye Lee, Min-Woo Lee

**Affiliations:** a Department of Integrated Biomedical Science, Soonchunhyang University, Cheonan, 31151, South Korea; b Soonchunhyang Institute of Medi-bio Science (SIMS), Soonchunhyang University, Cheonan, 31151, South Korea

## Abstract

Maintaining redox balance is crucial for mitochondrial homeostasis. During browning of white adipocytes, both the quality and quantity of mitochondria undergo dramatic changes. However, the mechanisms controlling the redox balance in the mitochondria during this process remain unclear. In this study, we demonstrate that thermogenic activation occurs before mitochondrial biogenesis during cold-induced browning of inguinal white adipose tissue (iWAT) and is accompanied by increased mitochondrial stress and integrated stress response (ISR) signaling. Specifically, cold exposure enhances the expression of ATF4, an ISR effector. Adipocyte-specific deletion of ATF4 results in increased energy expenditure, but paradoxically leads to a lower core body temperature, and heightened pro-inflammation in iWAT after cold exposure, which is restored by the antioxidant, MitoQ. Mechanistically, ATF4 regulates the redox balance through MTHFD2, an enzyme involved in mitochondrial redox homeostasis by NADPH generation. Cold exposure upregulates MTHFD2 expression in an ATF4-dependent manner, and its inhibition by DS18561882 *in vivo* leads to impaired cold-induced mitochondrial respiration similar to the effects of ATF4 loss. These findings suggest that ATF4 is essential for redox balance via MTHFD2, thereby affecting tissue homeostasis during iWAT browning.

## 1 Introduction

Endotherms have evolved sophisticated mechanisms to generate heat and maintain a stable body temperature across a wide range of environmental conditions, which are critical for cellular function and overall physiological stability [1,2]. Central to this thermal regulation is thermogenesis, driven by brown and beige adipocytes, which not only help sustain normal body temperature but also play a significant role in systemic energy expenditure. Accumulated evidence has underscored the vital role of adipose thermogenesis, highlighting its potential to alleviate metabolic disturbances by enhancing energy expenditure [[3], [4], [5], [6]].

Brown and beige adipocytes facilitate thermogenesis through uncoupling protein 1 (UCP1), which enables protons to leak across the inner mitochondrial membrane and transforms respiration from ATP production (coupled) to heat (uncoupled) [7,8]. Classical brown adipocytes consistently display thermogenic characteristics, including a high number of mitochondria and UCP1 expressions. In contrast, beige adipocytes are recruited and activated within white adipose tissue (WAT) in response to cold or cold-mimicking conditions, such as treatment with CL-316243, in a process known as "browning" [9]. During this transformation, white adipocytes undergo mitochondrial adaptations marked by increased expression of thermogenic genes, such as *Ucp1*, as well as enhanced mitochondrial respiration, biogenesis, and metabolic reprogramming [9,10]. Understanding the mechanisms regulating mitochondrial adaptation during the transition from white to beige adipocytes is crucial for effectively harnessing thermogenesis to combat metabolic diseases.

Reactive oxygen species (ROS) are important signaling molecules that facilitate cellular signaling and adaptation to stressors [11,12]. However, excessive ROS levels due to an imbalance between ROS production and antioxidant defenses can lead to substantial oxidative stress, which is closely linked to chronic metabolic diseases, such as diabetes, cancer, atherosclerosis, and cardiovascular disorders [[13], [14], [15], [16]]. Mitochondria, particularly complexes I and III of the electron transport chain, are the major sources of ROS. These organelles are particularly vulnerable to ROS toxicity because their components, including lipids and proteins, are directly exposed to ROS without protective barriers [17,18]. To mitigate ROS-induced damage, several antioxidant defense mechanisms are employed to mitigate ROS-induced damage: 1) enzymatic and non-enzymatic antioxidants, such as superoxide dismutase (SOD), peroxidase, glutathione peroxidase, catalase, metal-binding proteins (MBPs), glutathione, and coenzyme Q, directly regulate ROS levels [[19], [20], [21]]; and 2) enhanced uncoupled respiration or proton leak indirectly reduces ROS by decreasing mitochondrial activity [22,23]. Recent studies have also indicated that one-carbon metabolism in the mitochondria contributes to redox homeostasis through NADPH production, regulating ROS levels via GSH or thioredoxin reduction under hypoxic conditions and during tumor growth [[24], [25], [26]].

Emerging evidence underscores the importance of redox homeostasis in the cold-induced thermogenesis of adipose tissues. Cold exposure stimulates mitochondrial ROS production in brown adipose tissue (BAT), thereby enhancing its thermogenic activity. Chouchani et al. found that the accumulation of ROS enhances UCP1 activity through sulfenylation of the Cys-253 amino acid residue in UCP1 during thermogenic activation in BAT [27]. Additionally, a lipid byproduct of ROS, 4-hydroxy-2-nonenal (4-HNE), indirectly activates UCP1 by increasing proton conductance through fatty acid binding [28,29]. Accumulation of ROS may further contribute to the activation of BAT thermogenesis. Mice lacking Gpx1 or MnSOD showed elevated superoxide levels but were protected from insulin resistance induced by a high-fat diet, exhibiting enhanced uncoupled respiration and increased overall energy expenditure [30,31]. These findings indicated that ROS are essential for the regulation of cold-induced thermogenesis in adipocytes.

To adapt to environmental stress, cells employ a conserved intracellular signaling pathway known as the integrated stress response (ISR). Activation of ISR by various stressors, such as endoplasmic reticulum (ER) unfolded protein stress, oxidative stress, and amino acid deprivation, helps restore cellular homeostasis by regulating the transcription of adaptive response genes [32,33]. A key player in ISR is activating transcription factor 4 (ATF4), a basic leucine zipper transcription factor that binds to cAMP-responsive elements (CREs) or C/EBP-ATF response elements to modulate the transcription of target genes. ATF4 activation occurs through the phosphorylation of eIF2α, which reduces global protein synthesis while selectively enhancing ATF4 translation [34]. Two recent studies indicated that ATF4 regulates the thermogenic activity of BAT by managing amino acid pools, either dependent or independent of FGF21 function [35,36]. However, the role of ATF4 in the cold-induced browning of white adipose tissue remains unclear.

Enhanced mitochondrial respiration is a key feature of thermogenesis in the adipose tissue. Browning of white adipocytes involves substantial changes in both the quantity and quality of mitochondria [37]. Beige adipocytes likely possess adaptive mechanisms to manage mitochondrial stress and ensure optimal mitochondrial function to support thermogenesis and maintain adipose tissue homeostasis. Here, we suggest that ATF4 plays an important role in regulating redox balance through MTHFD2 shortly after cold exposure, thereby influencing mitochondrial and tissue homeostasis during adipose tissue browning.

## 2 Material and methods

### 2.1 Mouse studies

All animal studies were conducted according to protocols and guidelines approved by the Institutional Animal Care and Use Committee of Soonchunhyang University. All animals were congenic to the C57BL/6 background and housed at 22 °C (unless otherwise indicated) under a 12-h light/dark cycle. C57BL/6 mice were purchased from Orient Bio.

Adipocyte-specific ATF4 knockout mice (Atf4^AKO^) were generated by crossbreeding Atf4^flox/flox^ mice and Adipoq^Cre^ mice. Atf4^fl/fl^ (Atf4^WT^) mice were used as a littermate controls. All mice were maintained on a C57BL/6 background. In the rescue experiment, vehicles (10 % DMSO, 40 % PEG400, 5 % Tween-80) or MitoQ (5 mg/kg, i.p. injection, MedChemExpress) were injected into Atf4^WT^ and Atf4^AKO^ mice housed at room temperature, followed by cold exposure at 5 °C. For MTHFD2 pharmacological inhibition experiments, C57BL/6 mice were orally administered either vehicle (5 % DMSO, 95 % corn oil) or DS18561882 (100 mg/kg, MedChemExpress) by gavage.

Body composition was determined by InAlyzer Dual Energy X-ray Absorption Meter (Medikors).

### 2.2 Analysis of FGF21 serum levels

Serum FGF21 levels were measured using the Mouse FGF21 SimpleStep Elisa Kit (Abcam) according to the manufacturer's instructions. Briefly, serum was added to a pre-coated plate along with the antibody cocktail, incubated at room temperature for 1 h, and then washed. TMB substrate was added, and absorbance was measured at 450 nm. FGF21 concentrations were calculated based on a standard curve generated using recombinant mouse FGF21.

### 2.3 Indirect calorimetry measurement

Energy expenditure and associated measurements were performed using TSE Phenomaster metabolic cages equipped with the climate chamber (TSE systems) at Soonchunhyang Biomedical Research Core-facility of Korea Basic Science Institute (KBSI), following the manufacturer's protocols. Mice were acclimatized to the metabolic cages for 2 days prior to data collection. The temperatures were set as described in the results. Oxygen consumption rate (VO_2_) and CO_2_ release rate (VCO_2_) were monitored every 2 min for each cage. The respiratory exchange rate (RER) was calculated as the ratio of VO_2_ and VCO_2_. Food intake was monitored using the Phenomaster food measurement module. Total activity was measured by the number of beam breaks along the x- and z-axes. Real-time core body temperature was measured using an RFID transponder chip (UID Identification Systems) injected intraperitoneally into the mice.

### 2.4 Quantitative reverse transcription polymerase chain reaction (qRT-PCR)

Total RNA was extracted from homogenized tissues using TRIzol reagent (Invitrogen, Thermo Fisher Scientific). The isolated RNA was reverse transcribed using ReverTraAce™ qPCR RT SuperMix (Toyobo), and the resulting cDNA was used for quantitative PCR on a Quant Studio 3 real-time PCR detection system (Applied Biosystems) with SYBR green reagent (Toyobo). Relative mRNA expression levels were determined using the 2^–ΔΔCt^ method, with *36B4* as the internal reference control. For quantification of mitochondrial DNA (mtDNA), total DNA was isolated from the interphase and lower phenol-chloroform phase saved during total RNA extraction step. mtDNA content was quantified by real-time PCR using specific primers targeting the genes *Nd1*, *Nd6*, and *Cytb*. Primer sequences for the targeted genes are listed in Supplementary Table 1.

### 2.5 Immunoblotting

Snap-frozen tissues were homogenized in modified RIPA buffer (420 mM NaCl, 1 % NP-40, 0.1 % SDS, 0.5 % sodium deoxycholate, 50 mM Tris, pH 7.5, and a protease inhibitor cocktail) using a TissueLyser II (Qiagen). Total protein (20–40 μg) was separated by SDS-PAGE and transferred to PVDF membranes. Membranes were then blocked, and incubated with the indicated primary antibodies, followed by HRP-conjugated secondary antibodies. Chemiluminescence was detected using SuperSignal West Pico (Thermo Scientific) and developed using an automatic film processor (CP1000; Agfa-Gevaert). Antibody sources are listed in Supplementary Table 2.

### 2.6 Flow cytometry

iWAT was digested in 1 mL of Collagenase I buffer (2 mg/mL, Worthington) buffer at 37 °C for 20–30 min. The homogenates were washed and filtered through a 100 μm cell strainer before immunostaining for flow cytometric analysis. Cell suspensions were washed with FACS buffer (PBS, 0.5 M EDTA, and 2.5 % FBS), pre-incubated with Fc receptor block (0.5 μg), and then stained with appropriate primary antibodies. Data were acquired using a FACSCanto flow cytometer (BD Biosciences) and analyzed using FlowJo Software. Antibody sources and dilutions are listed in Table S2.

### 2.7 Cell culture

iWATs were dissected and digested in 2 mL collagenase I buffer (2 mg/mL at 250 U/g; Worthington) and 30 mg/mL bovine serum albumin (BSA) in Dulbecco's modified Eagle's medium (DMEM) at 37 °C for 20–30 min. The homogenates were washed and filtered through a 100 μm cell strainer. After centrifugation at 1500×*g* for 5 min, the pellet containing stromal-vascular cells (SVCs) was collected and resuspended in DMEM supplemented with 10 % FBS and 1 % penicillin/streptomycin. Cells were maintained at 37 °C in a humidified 5 % CO_2_ incubator.

For white adipocyte differentiation, isolated SVCs were treated with a differentiation cocktail containing 2 μg/mL dexamethasone, 0.5 mmol/L 3-isobutyl-1-methylxanthine, 5 μg/mL insulin, and 0.5 μmol/L rosiglitazone. Two days after induction, cells were maintained in culture media supplemented with 5 μg/mL insulin, with media changes every two days. For beige adipocyte differentiation, 125 μmol/L indomethacin and 1 μmol/L 3,3′,5-triiodo-l-thyronine were additionally included in the induction cocktail. During the maintenance period, 1 μmol/L 3,3′,5-triiodo-l-thyronine, and 0.5 μmol/L rosiglitazone were added to the media.

To mimic cold exposure *in vitro,* differentiated white adipocytes were treated with 5 μM of CL316,243 (Sigma). For the MitoQ rescue experiment, 100 nM of MitoQ (MedChemExpress) was applied to differentiated white adipocytes derived from SVCs of iWAT from Atf4^WT^ and Atf4^AKO^ mice. For chemical inhibition of MTHFD2, 10 μM DS18561882 (MedChemExpress) was treated with or without CL316,243 in differentiated white adipocytes.

### 2.8 Oil Red O staining

Differentiated beige adipocytes were washed with PBS and fixed in 4 % paraformaldehyde for 30 min at room temperature. After fixation, cells were rinsed with 60 % isopropanol and stained with freshly prepared Oil Red O solution (0.3 % in isopropanol, diluted 3:2 with distilled water) for 15–30 min. Following staining, excess dye was removed by washing with water. Images were captured using a light microscope. For quantification, the retained dye was eluted with isopropanol, and absorbance was measured at 500 nm.

### 2.9 Construction of MTHFD2-HA-expressing lentiviral vectors and transduction to SVCs

PCR primers were designed based on the mouse *Mthfd2* mRNA sequence. The forward primer was 5′-ATGGCTTCAGTTTCCTTGTTG-3′ and the reverse primer was 5′-CTAGTTGGTGGCGACTCCG-3’. cDNA synthesized from total RNA extracted from differentiated beige adipocytes was used as the template for amplification. The resulting *Mthfd2* PCR product was cloned into the pLVX-EF1α-TEV-HA-IRES-Puro vector at the *Eco*RI and *Xba*I sites, resulting in the insertion of an HA tag at the C-terminus of MTHFD2.

To generate a doxycycline-inducible lentiviral vector expression MTHFD2-HA, the *Mthfd2*-HA fragment was re-amplified and subcloned into the pLVX-TRE3GS-hPGK-TetOne-SV40-Puro vector via homologous recombination using the EZ-cloning Kit (Enzynomics). All constructs were validated by restriction enzyme digestion and DNA sequencing.

Lentiviral particles were produced by transfecting HEK293T cells with the *Mthfd2*-HA construct along with packaging plasmids (psPAX2 and pMD2.G) using Lipofectamine 3000 (Invitrogen). After 72 h, viral supernatants were collected and concentrated using Lenti-X concentrator (Takara) and used to transduce target cells. For transduction, SVCs isolated from iWAT were incubated with viral supernatant supplemented with 1 μg/mL polybrene for 48 h, followed by replacement with white adipocytes differentiation medium mentioned before. Transgene expression was induced by doxycycline where applicable.

### 2.10 Metabolic pathway analysis using Nanostring nCounter® analysis

A total of 768 genes related to metabolic processes and signaling pathway were analyzed using the nCounter® Metabolic Pathways Panel. The panel included target gene along with positive controls, negative control, and housekeeping gene for quality control and normalization. For each sample, 50 ng of RNA was used, and hybridization reactions were performed according to the manufacturer's instructions. Unique target-probe complexes were detected and scanned by the nCounter® SPRINT Profiler analysis system. Raw data assessment, quality control, and normalization were conducted using the nSolver™ analysis software V4.0. Control and housekeeping genes were selected using nSolver™ normalization module and resulting normalized data were used for downstream analysis. Differential gene expression was determined using a threshold of log_2_ fold-change ≤ −0.585 or ≥0.585 and a p-value <0.05.

### 2.11 RNA sequencing (RNA-seq) analysis

RNA was extracted from the iWAT of Atf4^WT^ and Atf4^AKO^ mice using TRIzol (Invitrogen) and mRNAs was purified using the oligo clean and concentrator kits (Zymo Research). RNA libraries for RNA-seq were prepared using the DNBSEQ Eukaryotic Strand-specific mRNA library kit according to the manufacturer's protocols. Raw reads containing adapter sequences or low-quality sequences were filtered using SOAPnuke with following parameters: "-n 0.01 -l 20 -q 0.4 --adaMR 0.25 --polyX 50 –minReadLen 150" [38]. Clean reads were mapped to UCSC mm10 reference genome using STAR. Uniquely mapped reads were quantified by featureCounts (parameter: -largestOverlap -B –C) [39,40]. Read counts were normalized using the DESeq2 algorithm (pyDESeq2) [41], and genes with a fold change >1.5 and a p-value <0.05 were identified as differentially expressed genes (DEGs). Gene ontology (GO) enrichment analysis of DEGs was performed using g:Profiler (g:GOSt) with g:SCS threshold 0.05) [42]. Data were obtained from three independent biological replicates.

### 2.12 ATF4 target prediction/transcription factor (TF) enrichment analysis

ATF4 target gene prediction was performed by overlapping DEGs identified via Deseq2 analysis, with publicly available databases, including MitoCarta3.0 (an inventory of mammalian mitochondrial proteins and pathways) and Chip-Atlas (containing BED filed from archived ChIP-Seq datasets). ATF4 binding site enrichment in the promoter region (defined as 2.0 kb upstream of the transcription start site) of nine downregulated genes-shared among the three datasets was predicted using microTSS. Transcription factor enrichment analysis was conducted using the R package, PWMEnrich.

### 2.13 Nuclear enrichment and lysis of adipocyte for chromatin-immunoprecipitation (ChIP) assay

ChIP assay from white adipocyte were performed following a previously published protocol [43]. Nuclei were isolated using hypotonic buffer (10 mM Tris-HCl pH 7.5, 10 mM NaCl, 3 mM MgCl_2_, 0.1 % IGEPAL CA630) supplemented with 1X Protease Inhibitor Cocktail (Sigma-Aldrich). Nuclear fractions were fixed by adding formaldehyde (HCHO; from 37 % HCHO-10 % methanol stock, Thermo Fisher Scientific) to a final concentration of 1 % followed by gentle shaking for 7 min. Fixation was quenched with 125 mM glycine for 5 min. Cells were centrifuged at 440×*g*, 4 °C for 5 min and the pellet was resuspended in 500 μL of ice-cold PBS with Protease Inhibitor Cocktail. After centrifugation at 5.800×*g* for 5 min at 4 °C, the pellet was resuspended in lysis buffer (10 mM Tris-HCl pH 7.5, 10 mM NaCl, 3 mM MgCl_2_, 0.5 % IGEPAL CA630) with 1X Protease Inhibitor Cocktail and centrifuged at 5.800×*g* for 5 min at 4 °C. The nuclei were then resuspended in 1 mL of ice-cold SDS lysis buffer (1 % SDS, 10 mM EDTA, 50 mM Tris-HCl pH 8.0) containing 1X Protease Inhibitor Cocktail.

### 2.14 ChIP assay

Chromatin was sheared using a COVARIS S220 Focused-ultrasonicator to yield DNA fragments ranging from 200 to 700 base pairs. The sheared chromatin was then diluted 10-fold in immunoprecipitation (IP) dilution buffer (0.037 % Triton X-100, 0.00033 % SDS, 167 mM NaCl, 0.0377 EDTA, 0.566 mM Tris-HCl pH 8.0) supplemented with 1X Protease Inhibitor Cocktail. Immunoprecipitation was performed at 4 °C for 4 h using an anti- ATF4 antibody, with Rabbit mAb IgG isotype used as a negative control. Pierce Protein A/G magnetic beads were used to form immunocomplexes with the targeted protein. Immunocomplexes were washed three time with IP wash buffer 1 (1 % Triton X-100, 0.1 % SDS, 150 mM NaCl, 2 mM EDTA, 20 mM Tris-HCl pH 8.0), three time with IP wash buffer 2 (1 % Triton X-100, 0.1 % SDS, 500 mM NaCl, 2 mM EDTA, 20 mM Tris-HCl pH 8.0), three time with IP wash buffer 3 (0.25 M LiCl, 1 % IGEPAL, 1 % Na-deoxycholate, 1 mM EDTA, 10 mM Tris-HCl pH 8.0), and finally once with Tris-EDTA buffer. Immunocomplexes were eluted in 200 μL of elution buffer (1 % SDS and 0.1 M NaHCO_3_). Protein-DNA complexes were digested in 50 μg/mL Proteinase K (Zymo Research) and reverse cross-linked by incubation at 65 °C overnight. The DNA was then treated with RNase A (Thermo Fisher Scientific) and purified by Oligo Clean & Concentrator kit (Zymo Research). Immunoprecipitated DNA was analyzed by real-time PCR using promoter-specific primers (sequences are listed in Supplementary Table S1).

### 2.15 Purification of intact mitochondria

Intact mitochondria were purified using the Mitochondrial Isolation Kit (Abcam), according to manufacturer's instruction. iWAT collected from wild-type mice was homogenized in Pre-filled Tube Kits I (Biocode) using BeadBug™ microtube homogenizer. The homogenates were centrifuged at 700×*g* for 10 min at 4 °C. The supernatant was transferred to a new tube and centrifuges at 10.000×*g* for 30 min at 4 °C. The purified mitochondrial pellet were resuspended in MAS buffer (70 mM sucrose, 220 mM mannitol, 5 mM KH_2_PO_4_, 2 mM MgCl_2_, 2 mM HEPES, 1 mM EGTA) supplemented with protease inhibitor cocktail. Protein concentration was determined using the BCA Assay (Thermo Fisher Scientific).

### 2.16 Mitochondria respirometry assay

Mitochondria respirometry was performed as previously described [44] using Seahorse XF96 Extracellular Flux analyzer (Agilent Technologies). 20 μg purified mitochondria were seeded into Poly-d-lysine-coated Seahorse XF96 microplate in 20 μl volume containing substrates. After centrifugation at 2000×*g* for 20 min at 4 °C, an additional 160 μl of MAS buffer + substrate were added to each well. For basal respiration, the substrate concentrations in the wells were 10 mM pyruvate +5 mM malate. To assess UCP1-mediated respiration, 3 mM GDP was injected with 40 μM Palmitoyl-l-carnitine +1 mM malate.

For *in vitro* mitochondria respirometry assay, SVCs derived from iWAT were seeded onto 96-well Seahorse XF96 microplate at 15,000 cells per well. On day 8 of differentiation, cells were rinsed once and the medium was replaced with 180 μL basal DMEM (Agilent Technologies) containing 25 mM glucose, 0.5 mM sodium pyruvate, 2 mM l-glutamine, and 1 % fatty acid-free BSA. After 1 h at 37 °C in a non-CO_2_ incubator, plates were inserted into a Seahorse XF96 Extracellular Flux analyzer. After three basal measurements, oligomycin was injected for a final concentration of 1.5 μM to inhibit ATP synthesis. FCCP was injected for a final concentration of 10 μM to determine maximal respiratory capacity. Finally, a mixture of rotenone and antimycin was injected to inhibit all mitochondrial respiration (1 μM each). Calculation of basal, maximum and proton leak oxygen consumption rate (OCR) was conducted based on report generator from Agilent Technologies.

### 2.17 NADP+/NADPH colorimetric assay

NADP+/NADPH levels were measured using NADP/NADPH Assay Kit (Abcam) following the manufacturer's instructions. Differentiated white adipocytes were treated with control or CL316,243 (5 μM) for 6 h. Cells were washed with cold PBS and scraped in 500 μL extraction buffer. The samples were subjected to two freeze/thaw cycles (20 min on dry ice, followed by 10 min at room temperature). The samples were vortexed for 10 s, centrifuged at 14,000×*g* for 5 min, and maintained on ice. For tissue measurements, approximately 50 mg iWAT were homogenized using Dounce homogenizer in 400 μL of an extraction buffer. The samples were transferred to a tube and centrifuged at top speed for 5 min. Additionally, 10 kD spin columns were used to remove enzymes that consume NADPH. The NADP+/NADPH assay (Abcam) was performed according to the manufacturer's protocol.

### 2.18 Quantification of GSH/GSSG

GSH and oxidized glutathione (GSSG) levels were assessed using a GSH/GSSG Ratio Detection Assay Kit (Abcam) following the manufacturer's instructions. For *in vitro* measurements, differentiated white adipocytes were treated with CL316,243 (5 μM), with or without DS18561882 (μM), for 6 h. Cells were washed with cold PBS and scraped in 100 μL mammalian buffer (PBS/0.5 % NP-40). For *in vivo* measurements, approximately 50 mg of iWAT were homogenized with Dounce homogenizer in 500 μL of a mammalian buffer. Samples were centrifuged for 15 min at 4 °C at top speed using a cold microcentrifuge. Spin columns (10 kDa) were used to remove enzymes that interfered with the analysis. The GSH assay mixture was added to the lysates and incubated for 60 min in the dark. Fluorescence was monitored at excitation/emission wavelengths of 490/520 nm.

### 2.19 Cellular and mitochondrial ROS detection

Total cellular and mitochondrial ROS levels were determined using 2′,7′-dichlorofluoresceindiacetate (DCFDA) and MitoSox™ Red (Thermo Scientific), respectively, according to the manufacturer's instructions. Differentiated white adipocytes were incubated with 10 μM DCFDA or 5 μM MitoSox™ Red for 20 min at 37 °C in the dark. After incubation, cells were washed with PBS, and fluorescence images were acquired using a Leica DMi8 microscope (Leica). Cells treated with TBHP- and FCCP served as positive controls for total cellular and mitochondrial ROS, respectively.

### 2.20 Histology

All tissues were fixed in 10 % formalin for 24 h after harvest, followed by immersion in 20 % sucrose in PBS at 4 °C until processing. The tissues were processed, embedded in paraffin, sectioned, stained with hematoxylin and eosin (H&E). Immunohistochemistry for UCP1 was performed using mouse and rabbit specific HRP/DAB detection IHC kit (Abcam) according to the manufacturer's instructions. Slides were scanned using Motic EasyScan One and images were acquired using DSAssistant (Motic).

### 2.21 Statistical analysis

Data were analyzed using Graph Pad Prism 8 software (GraphPad) and are presented as mean ± SEM. Statistical significance was determined using the unpaired two-tailed Student's *t*-test for single variables and two-way analysis of variance (ANOVA) followed by Sidak posttests for multiple variables. A p-value of <0.05 was considered statistically significant and is presented as ∗ (p < 0.05), ∗∗ (p < 0.01), or ∗∗∗ (p < 0.001).

## 3 Results

### 3.1 Thermogenic activation precedes mitochondrial biogenesis during browning of iWAT

Browning of white adipocytes involves the upregulation of thermogenic genes and the induction of mitochondrial biogenesis. To investigate the regulation of these processes during the browning of iWAT, we exposed 10-week-old C57BL/6 (WT) mice to a cold environment (5 °C) for 12 h, 24 h, 3 days, and 7 days, and analyzed thermogenic activity and mitochondrial biogenesis in iWAT. At the transcriptional level, the expression of both thermogenic and mitochondrial OXPHOS genes increased similarly between 12 and 24h of cold exposure (Supplementary Fig. 1a and b). However, at the translational level, the timing of protein expression differed; UCP1 protein levels increased as early as 12 h, reflecting the transcriptional changes, whereas mitochondrial OXPHOS proteins did not increase until 3 days after cold exposure (Supplementary Fig. 1c). Additionally, mitochondrial mass, assessed by the ratio of mitochondrial DNA to nuclear DNA (Supplementary Fig. 1d), and mitochondrial protein concentration (Supplementary Fig. 1e), increased after 3 days of cold exposure. Since UCP1 expression increased during the early period of WAT browning, we next examined whether mitochondrial respiration was altered. Ex vivo oxygen consumption analysis of purified iWAT mitochondria at 24 h and 3 days post cold exposure revealed early elevation of UCP1-dependent thermogenic activity (Supplementary Fig. 1f), suggesting that thermogenic activation precedes mitochondrial biogenesis during cold-induced browning of white adipocytes.

### 3.2 Cold exposure induces mitochondrial stress and activation of ATF4-mediated ISR

**Fig. 1:**
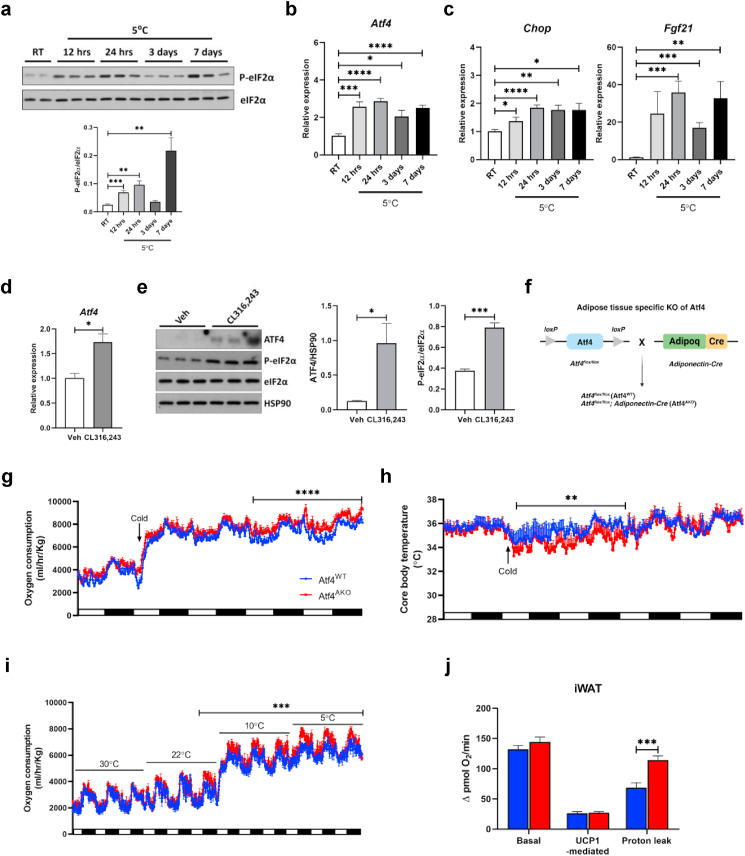
**Cold exposure activates the ATF4-mediated ISR, which is essential for maintaining systemic energy expenditure. (a**–**c)** Representative immunoblot analysis of phosphorylation of eIF2α (left) and densitometric quantification of phosphorylated eIF2α normalized to total eIF2α (right) **(a)**, quantitative RT-PCR analysis of *Atf4***(b)** and *Fgf21* and *Chop***(c)** in iWAT of 10-week-old C57BL/6 mice housed in RT or 5 °C for the indicated times (n = 5 per time point). Quantitative RT-PCR analysis of *Atf4***(d)** and immunoblot analysis of ATF4 and phosphorylated eIF2α (left), with densitometric quantifications of ATF4 normalized to HSP90, and phosphorylation of eIF2α normalized to total eIF2α (right) **(e)** in white adipocytes treated with CL-316,243 for 6 h (n = 3 per treatment). **(f)** Graphical scheme of the generation of adipocyte-specific ATF4 knockout mice. **(g and h)** Assessment of oxygen consumption (n = 6 per genotype) **(g)** and core body temperature (n = 5 for Atf4^WT^; n = 4 for Atf4^AKO^) **(h)** in 10-week-old Atf4^WT^ and Atf4^AKO^ mice housed at different ambient temperatures. **(i)** Assessment of cold-induced changes of oxygen consumption in 10-week-old Atf4^WT^ and Atf4^AKO^ mice (n = 3 per genotype). **(j)** Oxygen consumption rate in mitochondria isolated from iWAT as measured using the Seahorse XF96 Extracellular Flux analyzer (n = 6 per each group). Data are presented as mean ± SEM. ∗∗∗*p* < 0.005, ∗∗*p* < 0.01, ∗*p* < 0.05.

We previously demonstrated that phosphorylation of eIF2α inhibits translation of mitochondrial OXPHOS subunits in BAT [45]. To investigate whether the discordance between transcription and translation of mitochondrial OXPHOS subunits during iWAT browning is associated with eIF2α signaling, we examined eIF2α phosphorylation. Western blot analysis revealed increased phosphorylation of eIF2α up to 24 h after cold exposure (Fig. 1a), coinciding with the period of mismatch between mRNA and protein levels of mitochondrial OXPHOS subunits. Additionally, the expression of *Atf4*, a key downstream protein of eIF2α, along with its target genes, *Chop* and *Fgf21*, was upregulated at 12 h post-cold exposure (Fig. 1b and c). *In vitro* experiments further confirmed ISR activation during the browning of white adipocytes. Treatment of CL-316,243, a potent β3-adrenergic receptor agonist commonly used to induce thermogenesis [46], in white adipocytes differentiated from isolated iWAT SVC led to increased mRNA and protein levels of ATF4, as well as elevated phosphorylation of eIF2α (Fig. 1d and e), supporting ISR activation during iWAT browning.

Given that cold exposure robustly enhances mitochondrial respiration and the expression of mitochondria proteins, we next assessed the type of cellular stress activated during this period. Specifically, we examined stress responses related to mitochondria and the endoplasmic reticulum (ER). Mitochondrial stress-related chaperones, including *Hspe1*, *Hspd1*, and *Hsp70* were significantly upregulated after 24 h of cold exposure (Supplementary Fig. 1g). In contrast, ER stress-related chaperones, such as *Grp78* and the spliced-*Xbp1/*total-*Xbp1* ratio, remained unchanged (Supplementary Fig. 1h), indicating that mitochondrial stress, rather than ER stress, contributes to ISR activation. Taken together, these results suggest that ISR is activated by mitochondrial stress during the early stages of cold-induced WAT browning.

### 3.3 Loss of ATF4 in adipocytes impairs mitochondrial respiration by increasing proton leak

Since the ISR is activated early during iWAT browning in response to cold exposure, we investigated the role of ATF4 in this process. To this end, we generated adipocyte-specific ATF4 knockout mice (Atf4^AKO^) by crossing Atf4flox/flox (Atf4^WT^) mice with *Adiponectin*-Cre mice (Fig. 1f). While global deletion of ATF4 has been linked to developmental abnormalities and systemic metabolic defects [47,48], Atf4^AKO^ mice appeared phenotypically similar to their littermates (Supplementary Fig. 2a), with no significant difference in the morphology of iWAT, BAT, and epididymal WAT (Supplementary Fig. 2b). Body weight, body composition, and tissue masses were also comparable between genotypes (Supplementary Fig. 2c–e), suggesting that loss of ATF4 in adipocytes does not affect overall growth.

Given that BAT-specific deletion of ATF4 results in reduced core body temperature and impaired iWAT browning upon cold exposure [36], we next examined the metabolic phenotypes of adipocyte-specific ATF4 deletion using *Adiponectin*-Cre in response to cold exposure. To this end, we measured oxygen consumption in Atf4^WT^ and Atf4^AKO^ mice under cold conditions using metabolic cages. Atf4^AKO^ mice exhibited a significant increase in energy expenditure from day 3 onwards after cold exposure (Fig. 1g, Supplementary Fig. 2f). However, respiratory exchange ratio (RER), locomotor activity, and food intake were not significantly different between genotypes (Supplementary Fig. 2g–i). Core body temperature measurements revealed that Atf4^AKO^ mice had lower core body temperatures and required two days to recover to WT mice, indicating a greater energetic demand to main body temperature (Fig. 1h).

To confirm that the increased energy expenditure is due to the reduced environmental temperature, we acclimated Atf4^WT^ and Atf4^AKO^ mice to thermoneutral conditions (30 °C) for two weeks to suppress thermogenesis, then exposed to gradually colder temperatures (22 °C–10 °C to 5 °C). Under thermoneutral conditions, energy expenditure was similar between genotypes, but at lower temperature, Atf4^AKO^ mice displayed significantly higher energy expenditure (Fig. 1i, Supplementary Fig. 2j–m). These results suggest that the elevated energy expenditure in Atf4^AKO^ mice is triggered by reduced environmental temperature.

Next, we examined whether these changes in energy homeostasis are linked to mitochondrial activity by measuring oxygen consumption in isolated mitochondria from iWAT and BAT after 24 h of cold exposure. In iWAT mitochondria, ATF4 depletion did not alter basal or UCP1-mediated respiration but significantly increased proton leak (Fig. 1j). In contrast, BAT mitochondria showed no changes in basal respiration, UCP1-mediated respiration, or proton leak (Supplementary Fig. 3a). indicating that the metabolic alterations are specific to iWAT.

Given that respiratory changes were only observed in mitochondria of iWAT but not BAT, we assessed energy expenditure in mice of different ages to further evaluate the contribution of iWAT. Unlike BAT, which maintains thermogenic characteristics throughout life, beige adipose tissue are transiently present during early postnatal development, particularly around weaning at room temperature [8,9]. We hypothesized that if beige adipose tissue contributes to the increased energy expenditure in Atf4^AKO^ mice, younger mice may display more pronounced effects. Indeed, young Atf4^AKO^ mice (4.5–5.5 weeks old), which have a higher proportion of beige adipocytes, exhibited a rapid increased energy expenditure following cold exposure (Supplementary Fig. 3b–e). Moreover, no significant differences in BAT morphology (Supplementary Fig. 3f), UCP1 protein levels and *Ucp1* mRNA expression were observed between 10-week-old Atf4^WT^ and Atf4^AKO^ mice at room temperature or after 3 days of cold exposure (Supplementary Fig. 3g–i). Previous studies have proposed that ATF4 regulates cold-induced thermogenesis via modulation of amino acid turnover in BAT [35,36]. However, in our study, expression of amino acid metabolism-related genes in BAT remained unchanged following cold exposure (Supplementary Fig. 3i). Although *Fgf2*1 mRNA levels were significantly reduced in Atf4^AKO^ BAT (Supplementary Fig. 3j), serum FGF21 protein were not altered (Supplementary Fig. 3k), suggesting that BAT is unlikely to contribute to the observed phenotypes in Atf4^AKO^ mice.

### 3.4 Increased energy expenditure in Atf4^AKO^ mice is independent of beige adipogenesis

**Fig. 2:**
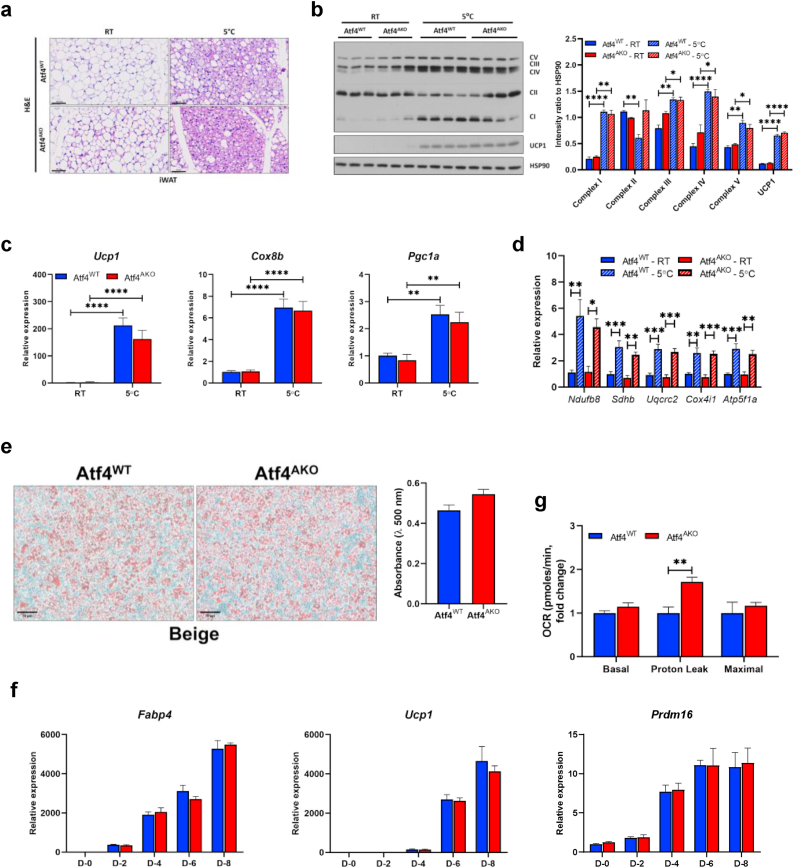
**Increased energy expenditure in Atf4^AKO^ mice is independent of beige adipogenesis. (a**–**d)** Representative images of Hematoxylin & Eosin (H&E) staining (scale bar, 60 μm) **(a)**, immunoblot analysis for mitochondria OXPHOS complexes and UCP1 (left), and densitometric quantification of OXPHOS complexes and UCP1 normalized to HSP90 (right) (n = 3 for each genotype housed in RT, n = 4 for each genotype housed in 5 °C) **(b)**, quantitative RT-PCR analysis of *Ucp1*, *Cox8b,* and *Pgc1a* (n = 5 per genotype) **(c)**, and quantitative RT-PCR analysis of OXPHOS complexes encoded genes (n = 5 per genotype) **(d)** in iWAT from 10-week-old C57BL/6 mice housed at RT or 5 °C for 3 days. **(e)** Oil red O (ORO) staining of beige adipocyte differentiated from SVCs isolated from iWAT of Atf4^WT^ and Atf4^AKO^ mice (scale bar, 70 μm; n = 3 per genotype). **(f)** Quantitative RT-PCR analysis of *Fabp4*, *Ucp1,* and *Prdm16* during beige adipogenesis of Atf4^WT^ and Atf4^AKO^ SVCs (n = 3 per genotype and time point). **(g)** Oxygen consumption rate of Atf4^WT^ and Atf4^AKO^ beige adipocytes, as measured using the Seahorse XF96 Extracellular Flux analyzer. Data are presented as mean ± SEM. ∗∗∗*p* < 0.005, ∗∗*p* < 0.01, ∗*p* < 0.05.

To determine whether the elevated energy expenditure in Atf4^AKO^ mice is attributable to enhanced beige adipogenesis in iWAT, we exposed both Atf4^WT^ and Atf4^AKO^ mice to cold temperatures (5 °C) for 3 days and assessed iWAT browning. Histological analysis revealed a modest increase in the number of multilocular adipocytes in the iWAT of Atf4^AKO^ mice at room temperature, a phenotype that persisted following cold exposure (Fig. 2a). Despite reduced ATF4 expression, UCP1 protein levels and thermogenic gene expression (*Ucp1, Cox8b,* and *Pgc1a*) were comparable between genotypes (Fig. 2b and c). Likewise, both mitochondrial OXPHOS gene expression and protein levels were similar in Atf4^WT^ and Atf4^AKO^ mice under both room temperature and cold condition (Fig. 2b–d). Consistent with these *in vivo* observations, *in vitro* experiments showed no significant differences in beige adipocyte differentiation. SVCs isolated from iWAT of both genotypes exhibited similar lipid droplet formation (Fig. 2e), and the expression of beige adipocyte differentiation marker genes, such as *Fabp4, Ucp1*, and *Prdm16*, remained unchanged throughout the differentiation process (Fig. 2f). However, seahorse analysis revealed a significant increase in proton leakage in ATF4-deficient beige adipocytes (Fig. 2g). These results suggest that the increased energy expenditure observed in Atf4^AKO^ mice is not driven by enhanced beige adipogenesis but rather by increased mitochondrial proton leak.

### 3.5 Depletion of ATF4 impairs the increase of type 2 immunity

**Fig. 3:**
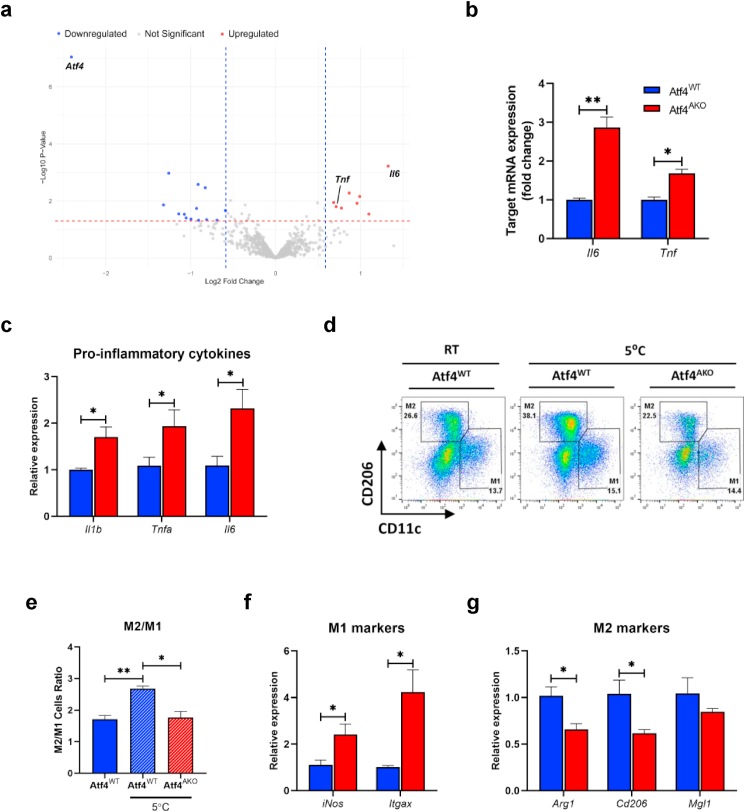
**ATF4 regulates macrophage homeostasis and inflammatory states in iWAT during cold exposure. (a)** Volcano plot analysis of differentially expressed genes from Metabolic Pathways Panel in iWAT from 10-week old Atf4^WT^ and Atf4^AKO^ mice housed at 5 °C for 3 days. Genes with a fold change >1.5 (red) or < - 1.5 (blue) and a P-value <0.05 are highlighted. **(b)** Relative normalized mRNA counts of *Il6* and *Tnf* from NanoString analysis. **(c**–**g)** Quantitative RT-PCR analysis of pro-inflammatory cytokines (*Il1b*, *Tnfa*, and *Il6*) **(c)**, representative flow cytometry **(d)**, M1 to M2 macrophage ratio **(e)**, quantitative RT-PCR analysis of M1 macrophage markers (*iNos* and *Itgax*) **(f)**, and M2 macrophage markers (Arg*1*, *Cd206*, *Mgl1*) **(g)** in iWAT from 10-week-old Atf4^WT^ and Atf4^AKO^ mice housed at RT or exposed to cold (5 °C) for 3 days (n = 5 per genotype). Data are presented as mean ± SEM. ∗∗∗*p* < 0.005, ∗∗*p* < 0.01, ∗*p* < 0.05.

To investigate the metabolic alterations caused by ATF4 depletion during iWAT browning, we analyzed the expression of metabolism-related genes in Atf4^WT^ and Atf4^AKO^ mice housed at room temperature or cold condition using the NanoString Mouse Metabolic Pathways Panel. A normalized heatmap showed that while cold exposure for 3 days altered metabolic gene expression such as *Pdk4*, *Ppargc1a*, *Cox10*, *Sod2*, and *Ndufs8*, these changes were independent of ATF4 depletion (Supplementary Fig. 4a, Supplementary Table 3). To explore the detailed alterations, we analyzed the differential gene expression of Atf4^WT^ mice and Atf4^AKO^ mice under cold conditions. A volcano plot showed significant changes in 8 upregulated and 14 downregulated genes in Atf4^AKO^ mice under cold conditions (Fig. 3a, Supplementary Fig. 4b). Notably, pro-inflammatory cytokines, such as *Il6* and *Tnf* were significantly upregulated in the absence of ATF4 (Fig. 3a and b). These findings were corroborated by quantitative real-time PCR (qRT-PCR), which revealed elevated expression of pro-inflammatory cytokines, including *Il1b, Tnfα,* and *Il6* in Atf4^AKO^ iWAT under cold stress (Fig. 3c). Given that cold-induced energy expenditure is closely correlated with enhanced type 2 immune response [49,50], we next examined whether ATF4 deletion alters immune cells composition during iWAT browning. Flow cytometry analysis revealed a significant increase in the M2/M1 macrophage ratio after cold exposure (Fig. 3d and e). However, Atf4^AKO^ mice failed to expand their M2 macrophage populations in response to cold stress (Fig. 3d), resulting in a lower M2/M1 macrophage ratio (Fig. 3e). This was further supported by the upregulation of M1 macrophage-associated genes, *iNos* and *Itgax* (Fig. 3f), and a reduction in M2 marker genes, such as Arg*1* and *Cd206* (Fig. 3g). Together, these results suggest that ATF4 depletion disrupts the establishment of a cold-induced type 2 immune response during iWAT browning.

### 3.6 ATF4 is required for maintaining mitochondrial ROS levels during cold exposure

**Fig. 4:**
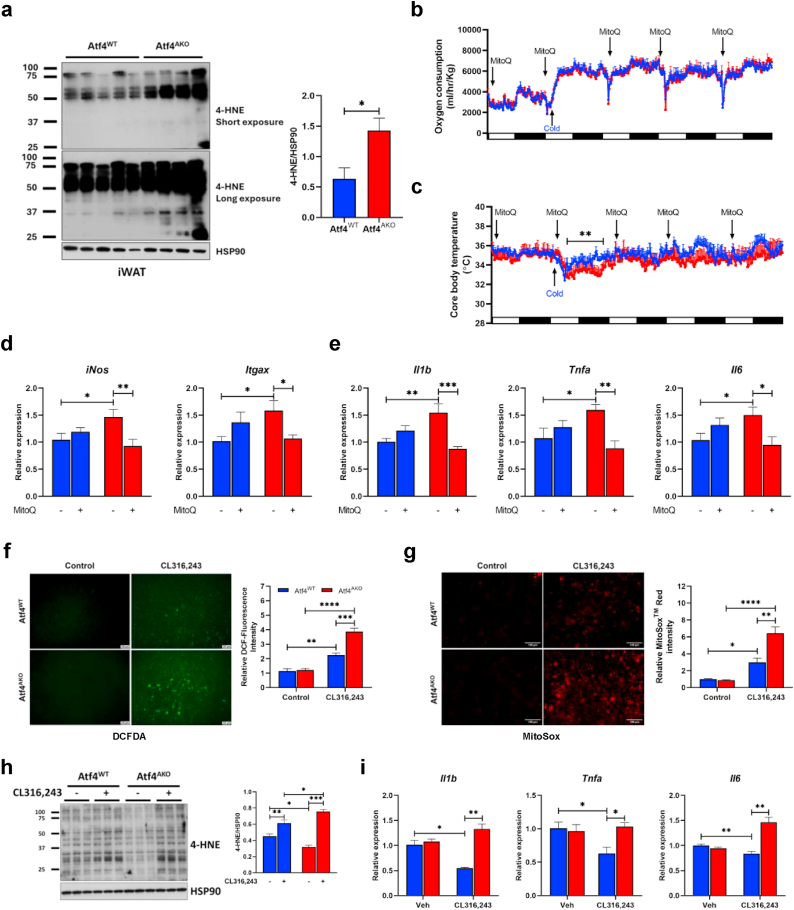
**ATF4 maintains mitochondrial ROS levels in adipocytes. (a)** Immunoblot analysis of 4-HNE (left) and densitometric quantification for 4-HNE normalized to HSP90 (right) in iWAT from 10-week-old Atf4^WT^ and Atf4^AKO^ mice exposed to cold (5 °C) for 24 h (n = 5 for Atf4^WT^; n = 4 for Atf4^AKO^). **(b**–**c)** Assessment of oxygen consumption **(b)** and core body temperature **(c)** in 10-week-old Atf4^WT^ and Atf4^AKO^ mice injected with MitoQ (5 mg/kg) during cold exposure (5 °C) (n = 4 per group). **(d and e)** Quantitative RT-PCR analysis of M1 macrophage markers (*iNos* and *Itgax*) **(d)** and pro-inflammatory cytokines (*Il1b*, *Tnfa*, and *Il6*) **(e)** in iWAT from 10-week-old Atf4^WT^ and Atf4^AKO^ mice exposed to cold (5 °C) for 3 days with or without injection of MitoQ (5 mg/kg) (n = 6 for Atf4^WT^ Vehicle treatment; n = 5 for Atf4^WT^ MitoQ treatment; n = 5 for Atf4^AKO^ Vehicle treatment; n = 6 for Atf4^AKO^ MitoQ treatment). **(f**–**i)** Fluorescence images and quantification of DCFDA staining **(f)** and MitoSox staining (scale bar, 100 μm; n = 3 per group) **(g)**, immunoblot analysis of 4-HNE (left) and densitometric quantification of 4-HNE normalized to HSP90 (right) (n = 3 per group) **(h)**, and quantitative RT-PCR analysis of pro-inflammatory cytokines (*Il1b*, *Tnfa*, and *Il6*) (n = 4 per group) **(i)** in Atf4^WT^ and Atf4^AKO^ white adipocytes treated with control or CL316,243 for 6 h. Data are presented as mean ± SEM. ∗∗∗*p* < 0.005, ∗∗*p* < 0.01, ∗*p* < 0.05.

Previous studies have shown that the accumulation of ROS contributes to an increased energy expenditure during cold exposure [27]. To investigate this, we examined the expression of 4-hydroxy-2-nonenal (4-HNE), a byproduct of lipid peroxidation widely recognized as a marker of oxidative stress. Western blot analysis revealed more accumulation of 4-HNE in the iWAT of Atf4^AKO^ mice after 24 h of cold exposure (Fig. 4a), whereas no such accumulation was observed in the BAT (Supplementary Fig. 5a). To assess whether ROS influence the altered mitochondrial respiration in Atf4^AKO^ mice, we treated both Atf4^WT^ and Atf4^AKO^ mice with MitoQ, a mitochondria-targeted derivative of the antioxidant ubiquinone and measured oxygen consumption. After MitoQ treatment, oxygen consumption, body weight, RER, locomotor activity, and food intake in Atf4^AKO^ mice were comparable to those in Atf4^WT^ mice (Fig. 4b, Supplementary Fig. 5b–f). Although Atf4^AKO^ mice treated with MitoQ exhibited a lower core body temperature in response to cold exposure, the drop was less pronounced and recovery occurred more rapidly—within approximately 24 h—compared to the untreated condition (Fig. 4c). In addition, MitoQ treatment had no effect on the expression of *Atf4* and *Ucp1* mRNA (Supplementary Fig. 5h). However, MitoQ significantly reduced the expression of *iNos* and *Itgax* (Fig. 4d) and *Il1b, Tnfα,* and *Il6* (Fig. 4e), which were upregulated by depletion of ATF4. These results suggest that ROS contribute to both increased energy expenditure and a pro-inflammatory state in Atf4^AKO^ mice during cold exposure.

Next, we investigated whether ATF4 depletion leads to increased ROS accumulation in adipocytes. ROS levels were assessed using DCFDA and MitoSox staining, which specifically detect ROS in the cytosol and mitochondria, respectively. CL-316,243 treatment induced DCFDA-positive cells in white adipocytes derived from the isolated SVCs of iWAT. This effect was more pronounced in ATF4-deficient adipocytes (Fig. 4f). MitoSox staining showed a similar trend, with a two-fold increase in ROS levels in ATF4- deficient adipocytes (Fig. 4g). The elevated ROS levels were corroborated by increased 4-HNE levels (Fig. 4h), suggesting that ATF4 depletion enhances mitochondrial ROS accumulation. Additionally, ATF4 depletion led to a significant increase in the expression of pro-inflammatory cytokines in adipocyte following CL-316,243 treatment (Fig. 4i). Given that ROS and 4-HNE modulate the mitochondrial respiration by activating UCPs or promoting proton leak [28,29,51], we examined whether elevated ROS levels in ATF4-deficient adipocytes contribute to increased mitochondrial proton leak. While basal and maximal mitochondrial respiration were not significantly altered in ATF4-deficient adipocytes after CL-316243 treatment (Supplementary Fig. 6a and b), the absence of ATF4 significantly increased proton leak in **ATF4-deficient** adipocytes treated with CL-316,243 (Supplementary Fig. 6c). Moreover, MitoQ treatment restored the elevated proton leak and pro-inflammatory cytokine expression induced by ATF4 deletion in adipocytes (Supplementary Fig. 6d–g). Together, these findings highlight that ATF4 is required for maintaining mitochondrial ROS homeostasis. Its depletion exacerbates ROS accumulation, which impacts mitochondrial respiration and promotes a pro-inflammatory state in adipocytes during cold exposure.

### 3.7 ATF4 regulates MTHFD2 expression to maintain redox balance in response to cold exposure

**Fig. 5:**
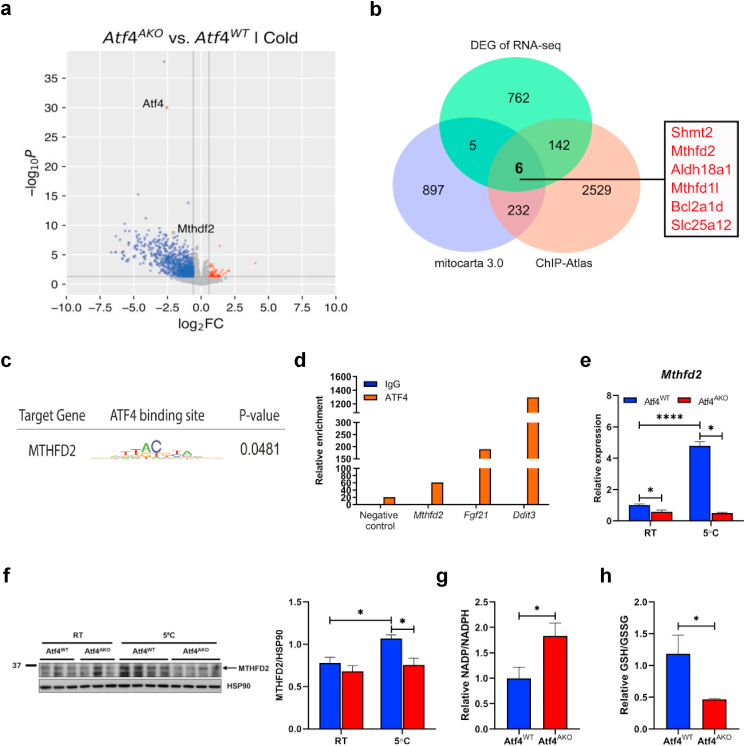
**ATF4 regulates MTHFD2 expressions and maintains redox balance. (a)** Transcriptomic analysis of iWAT from Atf4^WT^ and Atf4^AKO^ mice exposed to cold (5 °C) for 24 h. RNA-seq results are presented as a volcano plot. Genes with fold change >1.5 (red) or < −1.5 (blue) and p-value <0.05 are highlighted. **(b)** Venn diagram of genes from differentially expressed genes (DEG), Mitocarta 3.0, and ChIP-Atlas lists. Overlapping genes from the three lists are labeled in red. **(c)** Transcription factor enrichment analysis of promoter regions of genes labeled in red from Venn diagram shows significant enrichment of ATF4 binding sites in *Mthfd2*. **(d)** ChIP assay performed in differentiated white adipocytes; *Fgf21* and *Ddit3* were used as well-known ATF4 target genes. **(e**–**f)** Quantitative RT-PCR analysis of *Mthfd2* (n = 5 per genotype) **(e)** and immunoblot analysis of MTHFD2 (left) and densitometric quantification of MTHFD2 normalized to HSP90 (right) (n = 3 per genotype housed at RT; n = 4 per genotype exposed to cold) **(f)** in iWAT from 10-week-old Atf4^WT^ and Atf4^AKO^ mice housed at RT or exposed to cold (5 °C) for 3 days. **(g**–**h)** Ratios of NADP+/NADPH **(g)** and GSH/GSSG **(h)** in iWAT from 10-week-old Atf4^WT^ and Atf4^AKO^ mice exposed to cold (5 °C) for 3 days (n = 4 for Atf4^WT^; n = 5 for Atf4^AKO^). Data are presented as mean ± SEM. ∗∗∗*p* < 0.005, ∗∗*p* < 0.01, ∗*p* < 0.05.

To monitor the immediate response of ATF4 to cold exposure, we performed RNA-seq analysis on iWAT from Atf4^WT^ and Atf4^AKO^ mice housed in temperature or exposed to cold for one day. Consistent with previous findings, cold exposure significantly upregulated thermogenic genes such as *Ucp1*, *Ppargc1a*, and *Cox8b* in both genotypes (Supplementary Fig. 7a). Gene ontology biological process (GO:BP) enrichment analysis revealed that pathways related to thermogenesis—such as cellular respiration, mitochondrial gene expression, mitochondrial transport, mitochondrial organization—were among the most significantly upregulated in both Atf4^WT^ and Atf4^AKO^ mice following cold exposure (Supplementary Fig. 7b and c), suggesting that core components of the thermogenic and mitochondrial transcriptional program are induced independently of ATF4. To investigate ATF4-specific changes in gene expression under cold conditions, we analyzed differentially expressed genes (DEGs) between iWAT of Atf4^WT^ and Atf4^AKO^ mice under cold exposure. Total 915 DEGs were identified in Atf4^AKO^ group compared to Atf4^WT^ group, including 869 downregulated and 46 upregulated genes (fold change >1.5 or < −1.5, p-value <0.05) (Fig. 5a). We then sought to identify *ATF4* target genes involved in mitochondrial function. Using the Mitocarta 3.0 database, which lists 1140 mitochondria-related genes, and the ChIP-Atlas database, which predicts 2909 *ATF4* target genes, we intersected these with our DEGs, yielding 6 overlapping downregulated genes (Fig. 5b). To identify the direct targets of ATF4 among these 6 downregulated genes, we conducted transcription factor enrichment analysis of the 2 kb regions surrounding the transcription start sites (TSS). Among the 6 genes, MTHFD2 showed significant ATF4 enrichment in its TSS (Fig. 5c) and ChIP/qRT-PCR confirmed that ATF4 directly bound to the MTHFD2 promoter region (Fig. 5d). Furthermore, cold exposure robustly induced *Mthfd2* expression as early as 12 h post-exposure (Supplementary Fig. 8a), and this increase was significantly repressed in Atf4^AKO^ mice (Fig. 5e). Western blot analysis further confirmed that Atf4^AKO^ mice failed to induce MTHFD2 protein in response to cold exposure (Fig. 5f). We also examined other genes involved in one-carbon metabolism from the selected genes but found that *Mthfd1* and *Shmt2* expression remained unchanged in the absence of ATF4, indicating that MTHFD2 is specifically regulated by ATF4 during cold exposure (Supplementary Fig. 8b).

**MTHFD2** is essential for maintaining the redox balance in the mitochondria by producing NADPH, which is used to regenerate GSH from GSSG [24,52]. We found that cold exposure of Atf4^AKO^ mice led to an increased NADP+/NADPH ratio (Fig. 5g) and a decreased GSH/GSSG ratio (Fig. 5h) in iWAT. In **white adipocytes**, treatment with CL-316,243 for 6 h significantly increased the NADP+/NADPH ratio in the absence of ATF4 (Supplementary Fig. 8c), indicating compromised redox regulation at both tissue and cellular levels in the absence of ATF4. Notably, GSEA revealed that gene sets associated with ROS regulation were not significantly altered by ATF4 deletion in iWAT (Supplementary Fig. 8d). Consistently, qRT-PCR analysis confirmed that the expression levels of key antioxidant-related genes, including **Sod1*,* Sod2,** and **Gpx1,** were comparable between WT and ATF4-deficient adipocytes (Supplementary Fig. 8e).

We next investigated whether recovery of MTHFD2 would be sufficient to restore mitochondrial proton leak and reduce the pro-inflammatory state in Atf4^AKO^ adipocytes. To do this, we generated a doxycycline-inducible lentiviral vector by cloning *Mthfd2* into the pLV-Tet-On backbone. Using this lentiviral system, we induced MTHFD2 expression in white adipocytes differentiated from SVCs of Atf4^AKO^ mice. Gene expression analysis showed that ATF4-deficient adipocytes reduced *Mthfd2* expression compared to Atf4^WT^ adipocytes after CL316,243 treatment. Overexpression of MTHFD2 in Atf4^AKO^ adipocytes restored its expression similar to those in Atf4^WT^ adipocytes, without affecting *Atf4* or *Ucp1* mRNA expression (Supplementary Fig. 9a). Furthermore, overexpression of MTHFD2 reduced mitochondrial ROS accumulation in Atf4^AKO^ adipocytes treated with CL316,243, as evidenced by a reduction in MitoSox fluorescence signal (Supplementary Fig. 9b). Finally, MTHFD2 overexpression in Atf4^AKO^ adipocytes restored the increased proton leak (Supplementary Fig. 9c) and reduced pro-inflammatory cytokines expression (Supplementary Fig. 9d) following CL316,243 treatment. Together, these data suggest that MTHFD2 plays a key role in mediating ATF4 function, helping to maintain ROS homeostasis, mitochondrial proton leak, and an inflammatory state in adipocytes.

### 3.8 Pharmacological inhibition of MTHFD2 impairs mitochondrial respiration, lowers core body temperature and increases pro-inflammation

**Fig. 6:**
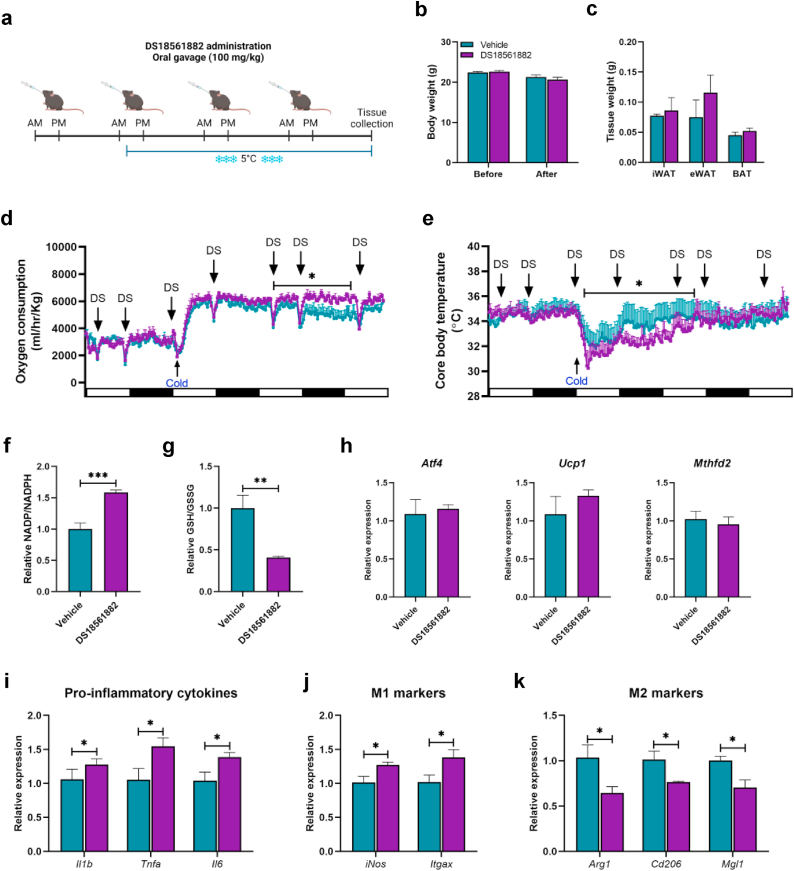
**Chemical inhibition of MTHFD2 leads to impaired energy homeostasis and inflammatory state *in vivo*. (a)** Graphical scheme of DS18561882 oral gavage administration (100 mg/kg). **(b**–**e)** Body weight **(b)** and tissue weight **(c)** before and after cold exposure, assessment of oxygen consumption **(d)**, and core body temperature **(e)** in 10-week-old C57BL/6 mice administered vehicle or DS18561882 (100 mg/kg) (n = 4 for vehicle; n = 5 for DS18561882). **(f**–**k)** Ratios of NADP+/NADPH **(f)** and GSH/GSSG **(g)**, quantitative RT-PCR analysis of *Atf4*, *Ucp1*, and *Mthfd2***(h)**, pro-inflammatory cytokines **(i)**, M1-macrophage markers **(j)**, and M2-macrophage markers **(k)** in iWAT from 10-week-old C57BL/6 mice administered vehicle or DS18561882 (100 mg/kg) (n = 4 for vehicle; n = 5 for DS18561882). Data are presented as mean ± SEM. ∗∗∗*p* < 0.005, ∗∗*p* < 0.01, ∗*p* < 0.05.

Next, we investigated whether MTHFD2 inhibition could mimic the phenotypic traits observed in Atf4^AKO^ mice. For this purpose, DS18561882, a selective MTHFD2 inhibitor, was administered orally twice daily to 10- week-old C57BL/6 mice, starting one day before cold exposure and continuing throughout the cold exposure period [53,54] (Fig. 6a). DS18561882 treatment did not affect mice body or adipose tissue weight (Fig. 6b and c). Metabolic analysis revealed that mice treated with DS18561882 had significantly higher energy expenditure compared to the vehicle-treated mice after 1.5 days of cold exposure (Fig. 6d). Notably, these mice also exhibited a lower core body temperature immediately following cold exposure, with delayed recovery, requiring approximately 1.5 days to reach a temperature similar to that of the vehicle group (Fig. 6e). In addition, DS18561882 treatment resulted in an elevated NADP+/NADPH ratio and a reduced GSH/GSSG ratio in iWAT (Fig. 6f and g), indicative of an alteration in redox homeostasis similar to that observed in Atf4^AKO^ mice. DS18561882 treatment had no effect on the expression of *Atf4*, *Ucp1*, and *Mthfd2* in iWAT (Fig. 6h). However, it significantly upregulated the expression pro-inflammatory cytokine genes (Fig. 6i), increased the expression of M1 macrophage-related genes (Fig. 6j), and decreased the expression of M2 macrophage-related genes (Fig. 6k).

**Fig. 7:**
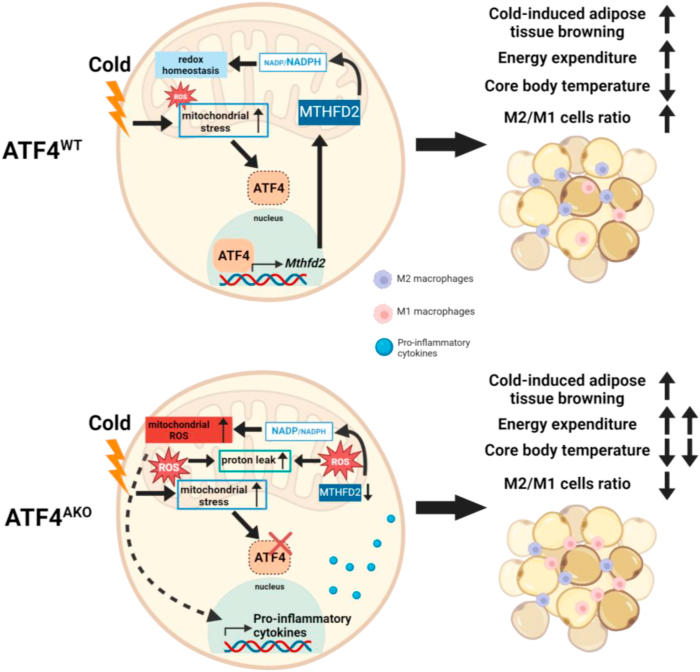
**Proposed model for the role of ATF4 during cold-induced browning**. ATF4 is rapidly activated in iWAT in response to acute cold exposure, where it plays a pivotal role in regulating MTHFD2 expression to maintain mitochondrial redox homeostasis. This regulation is essential for beige adipocyte function, facilitating systemic energy expenditure and preserving adipose tissue homeostasis during the browning of iWAT.

In *in vitro* experiments, co-treatment of CL316,243 and DS18561882 led to an increased NADP+/NADPH ratio (Supplementary Fig. 10d) and a decrease in the GSH/GSSH ratio (Supplementary Fig. 10e). MitoSox staining revealed that DS18561882 treatment significantly elevated mitochondrial ROS levels (Supplementary Fig. 10f). Immunoblot analysis revealed that DS18561882 treatment did not affect MTHFD2 protein expression in CL316,243-treated adipocytes (Supplementary Fig. 10g). Furthermore, the expression of pro-inflammatory cytokines was significantly increased (Supplementary Fig. 10h). Notably, **MTHFD2 inhibition** caused a greater proton leak than that induced by CL-316,243 alone in adipocytes, although it did not significantly affect basal or maximal mitochondrial respiration (Supplementary Fig. 10i–k). Taken together, these findings underscore the role of ATF4-MTHFD2 axis in maintaining mitochondrial redox balance, which subsequently influences mitochondria proton leak and pro-inflammatory state during the browning of iWAT (Fig. 7).

## 4 Discussion

The browning of white adipocytes during cold exposure is characterized by a marked transition from energy-storing cells to energy-dissipating cells. This process is associated with the upregulation of thermogenic genes, such as *UCP1*, increased mitochondrial respiration, and enhanced mitochondrial biogenesis [8,10]. Despite these extensive changes in mitochondrial quantity and quality, the mechanisms by which adipocytes maintain mitochondrial homeostasis during browning remain poorly understood. In this study, we provide evidence that ISR is activated early during cold exposure, with a particular focus on the transcription factor, ATF4, which is a key effector of ISR. Our data demonstrated that ATF4 is essential for the regulation of mitochondrial adaptation in adipocytes, specifically by regulating redox homeostasis through the enzyme MTHFD2. We observed that the depletion of ATF4 or inhibition of MTHFD2 activity led to higher ROS accumulation in adipocytes. Moreover, the inhibition of MTHFD2 by selective inhibitor treatment *in vivo* results in altered systemic energy regulation, which is characterized by an impaired mitochondrial respiration, required higher energy expenditure to maintain core body temperature. Additionally, the inhibition of MTHFD2 also exhibited a disruption in immune homeostasis in iWAT, as evidenced by heightened pro-inflammatory state such as increased gene expression of *Il1b*, *Tnfa*, and *Il6* and M1 macrophages in adipose tissue after cold exposure. Importantly, these phenotypes were reversed by alleviating oxidative stress with MitoQ treatment. Together, these findings suggest that ATF4 plays a role in regulating redox homeostasis and mitochondrial adaptation through MTHFD2 during the cold-induced browning of iWAT.

An important factor influencing mitochondrial respiratory activity is the regulation of oxidants and antioxidants, known as redox balance. ROS levels increase with increased mitochondrial activity, and maintaining appropriate ROS concentrations is crucial for sustaining mitochondrial function [55,56]. Previous studies have shown that cold exposure elevates ROS levels in brown adipocytes, leading to enhanced UCP1 activity or proton leakage and resulting in increased energy expenditure [27]. Unlike brown adipocytes, beige adipocytes emerge from browning of white adipocytes in response to cold exposure. This process significantly increases both mitochondrial respiration and mitochondrial mass. Although the redox balance is likely to be critical during the formation of beige adipocytes, there is currently no evidence on how the redox balance is regulated during this browning process. In this study, we demonstrated that ATF4 plays a key role in regulating the redox balance during the early browning of white adipocytes after cold exposure. When this regulation is impaired, the beige adipose tissue unlikely maintains its balance between energy metabolism and tissue remodeling. Specifically, in Atf4^AKO^ mice, we observed increased energy expenditure and promoted pro-inflammatory immune responses in the adipose tissue. This pro-inflammatory state is atypical for beige adipose tissue, as previous studies have consistently shown that cold exposure promotes an anti-inflammatory environment [49,57]. Our findings suggest that ROS accumulation is a factor in this discrepancy, as elevated ROS levels and the formation of 4-HNE, a lipid byproduct induced by ROS, were observed in iWAT and adipocytes from Atf4^AKO^. Moreover, the phenotypes observed in Atf4^AKO^ mice were fully recovered by treatment with the antioxidant, MitoQ. The significance of this redox balance by ATF4 lies in the adaptive response of adipose tissue to different stresses. While increased energy expenditure in Atf4^AKO^ mice during cold exposure could help reduce body weight, the persistent pro-inflammatory state may increase susceptibility to metabolic disturbances, especially when exposed to other stressors, such as a high-fat diet. Additionally, we found that ROS influenced thermogenic function in beige adipocytes. Although Atf4^AKO^ mice exhibited higher energy expenditure, they also exhibited lower core body temperatures after cold exposure. The existing literature suggests that ROS-induced increases in energy expenditure may occur through proton leakage, independent of UCP1-mediated heat production [31,58]. Notably, our ex vivo experiments demonstrated that iWAT of Atf4^AKO^ mice exhibited increased mitochondrial proton leak without changes in UCP1-mediated respiration following 24 h of cold exposure. The 4-HNE has been implicated in promoting proton leak through interactions with fatty acids. Therefore, the elevated 4-HNE levels observed in iWAT of ATF4-deficient mice and adipocytes may explain the discrepancy between energy expenditure and core body temperature.

Recent studies have highlighted the role of ATF4 in cold-induced thermogenesis within brown adipose tissue (BAT). Bjorkman et al. demonstrated that ATF4 expression in thermogenic adipocytes is essential for maintaining core body temperature and for the proper induction of thermogenic genes, including *Fgf21* and *Ucp1*, during cold exposure. However, while our study observed similar physiological phenotypes in mice with ATF4-depleted adipocytes, we noted distinct differences in the expression of thermogenic gene. This discrepancy likely arises from the differences between the two studies, including the Cre recombinase models employes and the specific environmental conditions under which cold exposure was conducted. The previous study used *Ucp1*-Cre mice to delete ATF4, while our study employed *Adipoq*-Cre mice model. In the adult mice, *Ucp1* expression is primarily confined to brown adipocytes, and thus *Ucp1*-Cre predominantly induces recombination in brown adipocytes, while *Adipoq*-Cre targets a broader population of mature adipocytes, including white, beige, and brown fat depots. It is plausible that, unlike *Ucp1*-Cre, the use of *Adipoq*-Cre results in the deletion of ATF4 from birth, potentially allowing for compensatory effects during growth, thus masking differences in thermogenic gene expression observed during cold exposure. However it is worth noting that *Ucp1* is also detectable in the iWAT of young mice housed in standard vivarium conditions (∼20–24 °C, mild cold), reflecting transient beige adipocyte activity during postnatal development [57]. Moreover, despite using *Ucp1*-Cre mice, the study by Paulo et al. also found no changes in thermogenic gene expression in iWAT [35]. In addition to genetic model differences, the conditions under which the mice were housed and exposed to cold may also explain the observed variations. Bjorkman et al. housed their mice at thermoneutrality (30 °C) for 3 days prior to cold exposure. This pre-conditioning likely further suppresses basal thermogenic gene expression while enhancing cold-induced transcriptional response. However, our mice were maintained at room temperature (∼24 °C) prior to cold exposure, a condition that may allow for partial beige adipocyte activity, potentially dampening the magnitude of cold-induced gene expression changes. Moreover, cold exposure in thermoneutral-housed mice may trigger a more robust cellular and systemic stress response, potentially activating a wider range of mechanisms including thermogenic programs.

Our findings suggest that ATF4 regulates redox balance via MTHFD2 during the browning of white adipocytes. MTHFD2 is a key protein involved in one-carbon metabolism in the mitochondria, with two main functions: purine synthesis and redox balance regulation [59,60]. In this study, we demonstrated that ATF4 directly enhanced MTHFD2 expression in response to cold exposure and that MTHFD2 expression was suppressed in Atf4^AKO^ mice. Reduced MTHFD2 levels in Atf4^AKO^ cells led to an increased NADP+/NADPH ratio, which impaired glutathione reduction and resulted in a higher GSSG/GSH ratio. This suggest that the ROS accumulation in Atf4^AKO^ mice was due to the decreased expression of MTHFD2. It is unclear whether purine concentration can affect increased energy expenditure in Atf4^AKO^ mice. Previous studies have shown that purines, such as ADP and GDP inhibit UCP1 activity [61,62]. Additionally, another research demonstrated that a reduction in MTHFD2 expression leads to lower purine levels [54], assuming that the decreased MTHFD2 expression in Atf4^AKO^ mice could potentially eliminate the inhibition of UCP1 activity, thereby promoting higher energy expenditure. However, it is unlikely that energy metabolism via purine synthesis is involved. First, there are conflicting metabolic pathways, as purine synthesis is an anabolic process, while cold-induced thermogenesis is driven by catabolic metabolism. Second, we observed no changes in the expression of *Shmt2* or *Mthfd1*, other proteins involved in one-carbon metabolism, in the Atf4^AKO^ mice. Third, purines are essential for mitochondrial and nuclear DNA replication, but there was no significant alteration in mitochondrial DNA content in Atf4^AKO^ cells, suggesting that purine concentration does not play a major role in modulating energy expenditure.

Although ISR has been extensively studied in the context of cellular stress regulation across various cell types [32,33], its physiological significance in beige adipocytes remains unclear. In the present study, we observed that thermogenic activation precedes mitochondrial biogenesis during the browning of white adipocytes in response to cold exposure. It is unclear why thermogenic activation and mitochondrial biogenesis are sequentially regulated during the early stages of iWAT browning. This sequential regulation may be crucial for efficient energy distribution, enabling an immediate response to fluctuating environmental temperatures. First, to survive a decrease in body temperature, organisms prioritize thermogenic activation by channeling all available energy into essential thermogenic machinery, such as UCP1, before increasing mitochondrial content. By maximizing thermogenic activation first, organisms effectively conserve energy during the initial cold response. Second, organisms prevent unnecessary energy expenditure in mitochondrial biogenesis or mitophagy in preparation for further temperature changes [63]. Both are an energy-intensive process that requires substantial resources for the complex, multi-step process involved. As shown in this study, the modulation of mitochondrial OXPHOS subunits at the translational level is suggested by the observed discrepancy between the timing of OXPHOS subunit mRNA expression and protein synthesis upon eIF2α phosphorylation. Specifically, a delay in protein translation can limit the immediate synthesis of mitochondrial components, thus prioritizing thermogenesis over mitochondrial expansion during the initial cold exposure phase. 10.13039/100014337Furthermore, our previous findings support this hypothesis, showing that persistent phosphorylation of eIF2α in brown adipocytes inhibits the mitochondrial translation of OXPHOS subunits, resulting in abnormal mitochondrial morphology and dysfunctional bioenergetics [45]. Considering the function of eIF2α and ATF4 as observed, ISR is required for iWAT browning via regulation of mitochondrial adaptation early in cold stimulation.

## 5 Conclusions

Our study revealed adaptive mechanisms controlled by ATF4 during the transition from white to beige adipocytes in response to cold exposure. We showed that mitochondrial stress occurs early during cold exposure, triggering the activation of ATF4-mediated ISR, which is crucial for maintaining the functional accumulation of mitochondrial ROS and redox balance through the regulation of MTHFD2 expression. Additionally, our findings highlight the importance of ATF4-mediated regulation of mitochondrial ROS in sustaining systemic energy expenditure and preserving immune homeostasis in the beige adipose tissue during cold stress. Given that adult humans have relatively low levels of constitutively active BAT, most of their thermogenic adipose tissue must be recruited and activated by prolonged cold exposure or pharmacological triggers [64]. Understanding the detailed molecular mechanisms driving cold-induced adipose tissue browning could offer valuable insights for the development of novel strategies to prevent or treat obesity and related metabolic disorders.

## 6 Limitations of the study

This study has several limitations. While our findings show that adipocyte-specific deletion of ATF4 disrupts redox homeostasis and induces a pro-inflammatory state in iWAT during cold exposure, it remains unknown whether these effects are reversible upon rewarming to room temperature or whether they increases vulnerability to metabolic dysfunction under additional stressors, such as high-fat diet feeding. Moreover, due to technical challenges in directly measuring ROS levels during cold-induced adipose tissue browning, we relied on 4-HNE, a lipid peroxidation byproduct, as an indirect indicator of ROS. Finally, although our data suggest that elevated ROS accumulation in ATF4-deficient adipocytes contributes to increased mitochondrial proton leak, the underling molecular mechanism are yet to be fully understood. Future investigations should address these unresolved questions.

## CRediT authorship contribution statement

**Rehna Paula Ginting:** Writing – original draft, Visualization, Validation, Methodology, Formal analysis, Data curation. **Hoang-Anh Pham-Bui:** Writing – original draft, Investigation, Formal analysis. **Choijamts Munkhzul:** Software, Formal analysis. **Siti Aisyah Fuad:** Writing – review & editing, Validation. **Ahyeon Son:** Software, Formal analysis. **Jong-Seok Moon:** Software, Resources. **Jaeseok Han:** Writing – review & editing, Supervision, Resources. **Mihye Lee:** Writing – review & editing, Supervision, Resources, Funding acquisition, Formal analysis. **Min-Woo Lee:** Writing – review & editing, Supervision, Investigation, Funding acquisition, Data curation, Conceptualization.

## Data availability

The raw RNA-seq data have been deposited in the GEO database under accession number GSE294416.

## Declaration of competing interest

The authors declare that they have no known competing financial interests or personal relationships that could have appeared to influence the work reported in this paper.

## Data Availability

Data will be made available on request.

## Appendix A Supplementary data

Fig. S1. **Thermogenic activation precedes mitochondrial biogenesis during cold-induced iWAT browning.** (a-e) Quantitative RT-PCR analysis of thermogenic genes **(a)** and mitochondria OXPHOS complex genes **(b)**, representative immunoblot analysis for UCP1 and OXPHOS complexes and densitometric quantification of UCP1 and OXPHOS complexes normalized to HSP90 (n = 5 per time point) **(c)**, ratio of mitochondrial to nuclear genomic DNA reflecting mitochondrial number (n = 5 per group) **(d)**, and total mitochondrial protein (n = 4 per group) **(e)** from iWAT of 10-week-old C57BL/6 male mice subjected to 5 °C for the indicated times. **(f)** Basal oxygen consumption rate (OCR) and UCP1-mediated OCR of mitochondria isolated from iWAT of 10-week-old C57BL/6 male mice subjected to 5 °C for the indicated times, as measured by using Seahorse XF96 Extracellular Flux analyzer (n = 4 per group). **(g**–**h)** Quantitative RT-PCR analysis of mitochondrial-stress-related genes (*Hspe1*, *Hspd1*, *Hsp70*) **(g)** and ER-stress-related genes (*Grp78*, *Herpud1*, and *sXbp1/tXbp1*) **(h)** in iWAT from 10-week-old C57BL/6 male mice subjected to 5 °C for the indicated times (n = 5 per group). Data are presented as mean ± SEM. ∗∗∗*p* < 0.005, ∗∗*p* < 0.01, ∗*p* < 0.05. Fig. S2. **Real-time metabolic assessment of Atf4^WT^ and** A**tf4^AKO^ mice**. **(a**–**e)** Representative images of Atf4^WT^ and Atf4^AKO^ mice **(a)**, iWAT, eWAT, and BAT **(b)**, body weight **(c)**, body composition **(d)**, and tissue weight **(e)** from Atf4^WT^ and Atf4^AKO^ mice at room temperature (n = 4 for Atf4^WT^; n = 5 for Atf4^AKO^). **(f**–**i)** Assessment of oxygen consumption (bar graph) **(f)**, RER **(g)**, total activity **(h)**, and food intake **(i)** in 10-week-old Atf4^WT^ and Atf4^AKO^ mice housed at different ambient temperatures (n = 6 per genotype). **(j**–**m)** Assessment of oxygen consumption (bar graph) **(j)**, RER **(k)**, total activity **(l)**, and food intake **(m)** in 10-week-old Atf4^WT^ and Atf4^AKO^ mice housed at various temperatures (n = 3 per genotype). Data are presented as mean ± SEM. ∗∗∗*p* < 0.005, ∗∗*p* < 0.01, ∗*p* < 0.05. Fig. S3. **Analysis of thermogenic function in BAT. (a)** Oxygen consumption rate in mitochondria isolated from BAT, as measured using the Seahorse XF96 Extracellular Flux analyzer (n = 6 per group). **(b**–**e)** Assessment of oxygen consumption **(b)**, RER **(c)**, total activity **(d)**, and food intake **(e)** in 4-5-week-old Atf4^WT^ and Atf4^AKO^ mice (n = 5 for Atf4^WT^; n = 4 for Atf4^AKO^). **(f**–**i)** Representative images of H&E staining (scale bar, 60 μm) **(f)**, immunohistochemistry of UCP1 (scale bar, 60 μm) **(g)**, immunoblot analysis of UCP1 (left) and densitometric quantification of UCP1 normalized to HSP90 (right) **(h)**, and quantitative RT-PCR analysis of *Atf4* and amino acid metabolism-related genes **(i)** in BAT from 10-week-old Atf4^WT^ and Atf4^AKO^ mice housed at RT or exposed to cold (5 °C) for 3 days (n = 5 for Atf4^WT^; n = 4 for Atf4^AKO^). **(j**–**k)** Quantitative RT-PCR analysis of *Fgf21* in BAT (n = 5 per group) **(j)**, and serum FGF21 levels (n = 4 per group) **(k)** in 10-week-old Atf4^WT^ and Atf4^AKO^ mice housed at RT or exposed to cold (5 °C) for 3 days. Data are presented as mean ± SEM. ∗∗∗*p* < 0.005, ∗∗*p* < 0.01, ∗*p* < 0.05. Fig. S4. Analysis of metabolic pathway in iWAT using NanoString. (a) Heatmap showing normalized pathway expression from the Metabolic Pathway Panel comparing iWAT from Atf4^WT^ and Atf4^AKO^ mice housed at room temperature or exposed to cold (5 °C) for 3 days. **(b)** List of genes showing the most significant differences in the Metabolic Pathway Panel between iWAT from Atf4^WT^ and Atf4^AKO^ mice exposed to cold for 3 days. Fig. S5. Effects of MitoQ treatment in Atf4^WT^ and Atf4^AKO^ mice exposed to cold. (a) Immunoblot analysis of 4-HNE (left) and densitometric quantification of 4-HNE normalized to HSP90 (right) in BAT from 10-week-old Atf4^WT^ and Atf4^AKO^ mice exposed to 24 h cold (n = 5 for Atf4^WT^; n = 4 for Atf4^AKO^). **(b)** Body weight of Atf4^WT^ and Atf4^AKO^ mice before and after MitoQ treatment during cold exposure. **(c**–**f)** Assessment of oxygen consumption (bar graph) **(c)**, RER **(d)**, total activity **(e)**, and food intake **(f)** in 10-week-old Atf4^WT^ and Atf4^AKO^ mice injected by MitoQ (5 mg/kg) during cold exposure (n = 4 per treatment). **(g)** Quantitative RT-PCR analysis of *Atf4* and *Ucp1* in iWAT of 10-week-old Atf4^WT^ and Atf4^AKO^ mice exposed to cold (5 °C) for 3 days with or without injection of MitoQ (5 mg/kg) (n = 6 for Atf4^WT^ Vehicle; n = 5 for Atf4^WT^ MitoQ; n = 5 for Atf4^AKO^ Vehicle; n = 6 for Atf4^AKO^ MitoQ). Data are presented as mean ± SEM. ∗∗∗*p* < 0.005, ∗∗*p* < 0.01, ∗*p* < 0.05. Fig. S6. MitoQ treatment restores imbalanced mitochondrial respiration and the pro-inflammatory state. (a-c) Mitochondrial basal respiration **(a)**, maximal respiration **(b)**, and proton leak **(c)** in Atf4^WT^ and Atf4^AKO^ white adipocytes treated with CL316,243 for 6 h, as measured using the Seahorse XF96 Extracellular Flux analyzer (n = 4 per group). **(d**–**f)** Mitochondrial basal respiration **(d)**, maximal respiration **(e)**, and proton leak **(f)** in Atf4^WT^ and Atf4^AKO^ white adipocytes treated with CL316,243 with or without MitoQ for 6 h, as measured using the Seahorse XF96 Extracellular Flux analyzer (n = 5 per group). **(g)** Quantitative RT-PCR analysis of *Il1b*, *Tnfa*, *Il6* in Atf4^WT^ and Atf4^AKO^ white adipocytes treated with CL316,243 with or without MitoQ for 6 h (n = 3 per group). Data are presented as mean ± SEM. ∗∗∗*p* < 0.005, ∗∗*p* < 0.01, ∗*p* < 0.05. Fig. S7. Cold exposure induces thermogenic and mitochondrial transcriptional programs in iWAT independent of ATF4. (a) Transcriptomic analysis of iWAT from 10-week-old Atf4^WT^ and Atf4^AKO^ mice housed at room temperature or exposed to cold (5 °C) for 24 h. RNA-seq results are presented as a volcano plot. Genes with a fold change >1.5 (red) or < −1.5 (blue) and a p-value <0.05 are highlighted. Cold exposure resulted in 2945 differentially expressed genes (DEGs) in iWAT from Atf4^WT^ mice, including 1390 downregulated and 1555 upregulated genes. In Atf4^AKO^ mice, 2306 DEGs were identified in iWAT, comprising 1177 downregulated and 1129 upregulated genes.**(b**–**c)** Gene ontology biological process (GO:BP) enrichment analysis of DEGs in iWAT from Atf4^WT^**(b)** and Atf4^AKO^**(c)** mice after 24 h of cold exposure. Fig. S8. ATF4 does not affect the expression of other one-carbon metabolism-related gene and antioxidant-related gene expressions. (a) Quantitative RT-PCR analysis of *Mthfd2* in iWAT of 10-week-old male wild-type mice subjected to 5 °C for the indicated times (n = 5 per group). **(b)** Quantitative RT-PCR analysis of *Mthfd1* and *Shmt2* in iWAT of 10-week-old Atf4^WT^ and Atf4^AKO^ mice housed at RT or exposed to cold (5 °C) for 3 days (n = 4 for Atf4^WT^ and n = 5 for Atf4^AKO^ mice housed in RT; n = 5 per genotype mice exposed to 5 °C). **(c)** Ratios of NADP+/NADPH in Atf4^WT^ and Atf4^AKO^ white adipocytes treated with control or CL316,243 for 6 h (n = 3 per group). **(d)** Gene set enrichment analysis (GSEA) of *reactive oxygen species biosynthetic process* and *response to reactive oxygen species* gene sets from RNA-seq data in iWAT from Atf4^WT^ and Atf4^AKO^ mice exposed to cold for 24 h. For the *reactive oxygen species biosynthetic process* gene set, the normalized enrichment score (NES) was −0.842, with false discovery rate (FDR) q-value of 0.9623 and a family-wise error rate (FWER) p-value of 0.998. For the *response to reactive oxygen species* gene set, the NES was −0.9754, with a FDR q-value of 0.8964 and a FWER p-value of 0.9595. **(e)** Quantitative RT-PCR analysis of antioxidant markers (*Sod1*, Sod2, and *Gpx1*) (n = 4 per group) in Atf4^WT^ and Atf4^AKO^ white adipocytes treated with control or CL316,243 for 6 h. Data are presented as mean ± SEM. ∗∗∗*p* < 0.005, ∗∗*p* < 0.01, ∗*p* < 0.05. Fig. S9. Overexpression of MTHFD2 restores ROS accumulation, mitochondria proton leak, and the pro-inflammatory state in Atf4^AKO^ adipocytes. (a-d) Quantitative RT-PCR analysis of *Atf4*, *Mthfd2,* and *Ucp1* (n = 3 per group) **(a)**, representative of MitoSox fluorescence images and quantification (scale bar, 100 μm; n = 5 per group) **(b)**, mitochondrial proton leak, as measured using the Seahorse XF96 Extracellular Flux analyzer (n = 5 per group) **(c)**, and quantitative RT-PCR analysis of *Il1b*, *Tnfa,* and *Il6* (n = 3 per group) **(d)** in Atf4^WT^ and Atf4^AKO^ white adipocytes treated with CL316,243 for 6 h, with or without MTHFD2 overexpression. Data are presented as mean ± SEM. ∗∗∗*p* < 0.005, ∗∗*p* < 0.01, ∗*p* < 0.05. Fig. S10. Chemical inhibition of MTHFD2 leads to increased ROS accumulation and impaired redox homeostasis in adipocytes. (a-c) Assessment of total activity **(a)**, RER **(b)**, and food intake **(c)** in 10-week-old C57BL/6 mice treated with vehicle or DS18561882 housed at 22 °C–5 °C (n = 4 for vehicle; n = 5 for DS18561882). **(d**–**e)** Ratio of NADP+/NADPH (n = 4 for vehicle; n = 3 for DS18561882) **(d)** and GSH/GSSG (n = 3 per group) **(e)** in white adipocytes treated with DMSO or DS18561882 in response to CL316,243. **(f**–**j)** Representative MitoSox fluorescence images and quantification (scale bar, 100 μm; n = 3 per group) **(f)**, immunoblot analysis of MTHFD2 (left) and densitometric quantification for MTHFD2 normalized to HSP90 (right) (n = 3 per group) **(g)**, quantitative RT-PCR analysis of pro-inflammatory cytokines (*Il1b*, *Tnfa*, and *Il6*) (n = 3 per group) **(h)**, mitochondrial basal respiration **(i)**, maximal respiration **(j)**, and proton leak **(k)** measured using the Seahorse XF96 Extracellular Flux analyzer (n = 8 per group) in white adipocytes treated with DMSO or DS18561882 in response to CL316,243. Data are presented as mean ± SEM. ∗∗∗*p* < 0.005, ∗∗*p* < 0.01, ∗*p* < 0.05.Multimedia component 1
