# Supplementary material for: Regulation of redox homeostasis by ATF4-MTHFD2 axis during white adipose tissue browning
Source: Redox Biol. 2025 Jun 9;85:103715. doi: 10.1016/j.redox.2025.103715 (PMC12205661; doi:10.1016/j.redox.2025.103715)

Supplementary Fig. 1

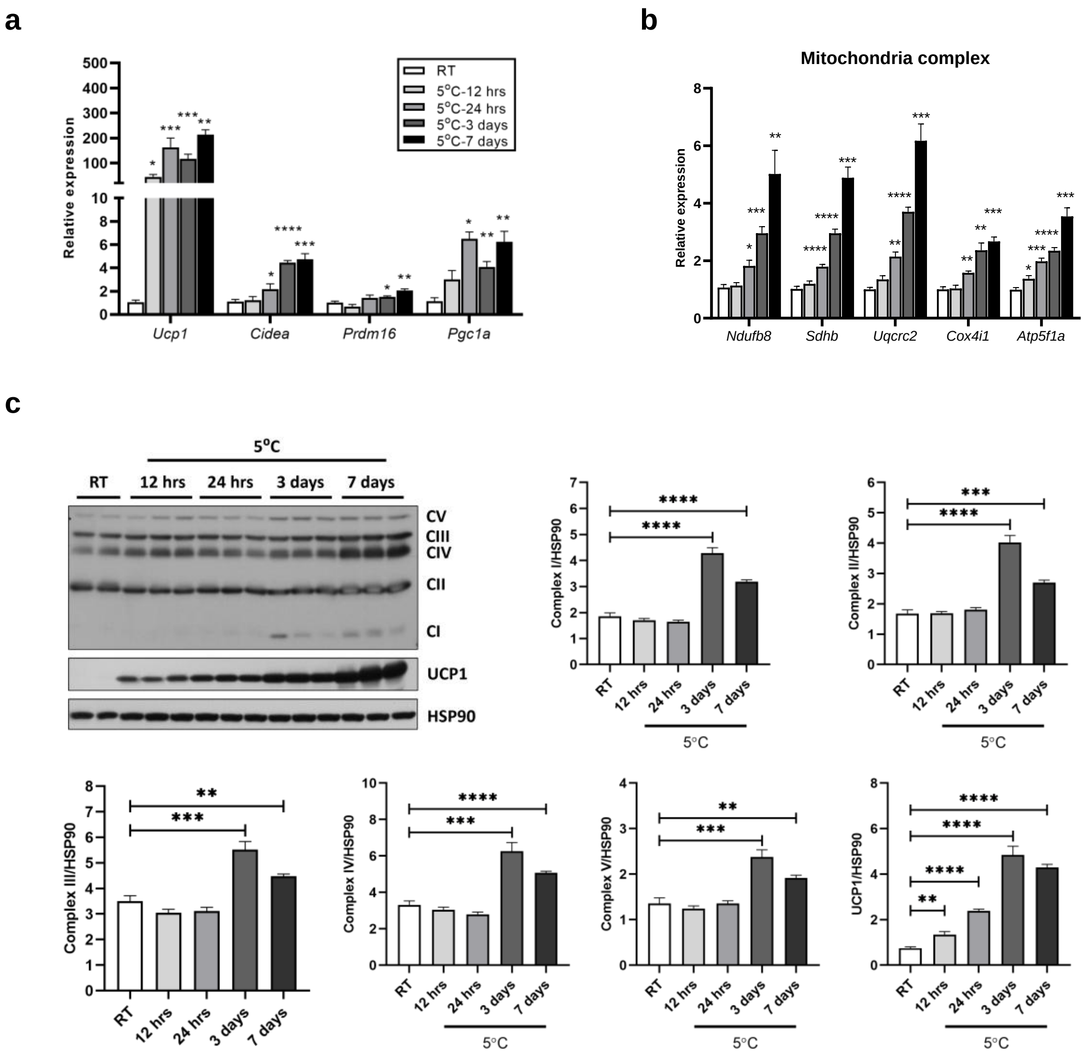

Supplementary Fig. 1 cont.

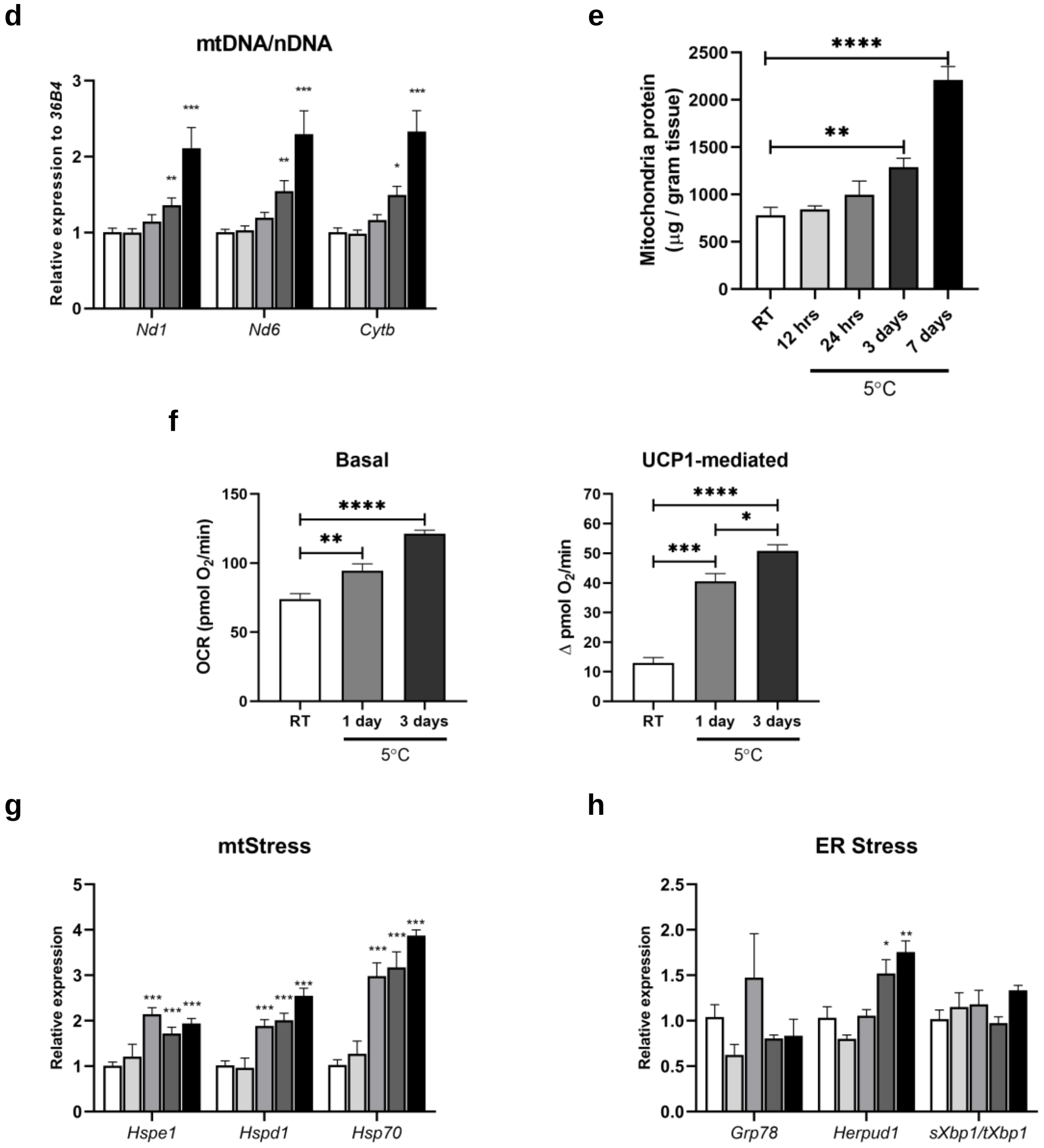

Supplementary Fig. 2

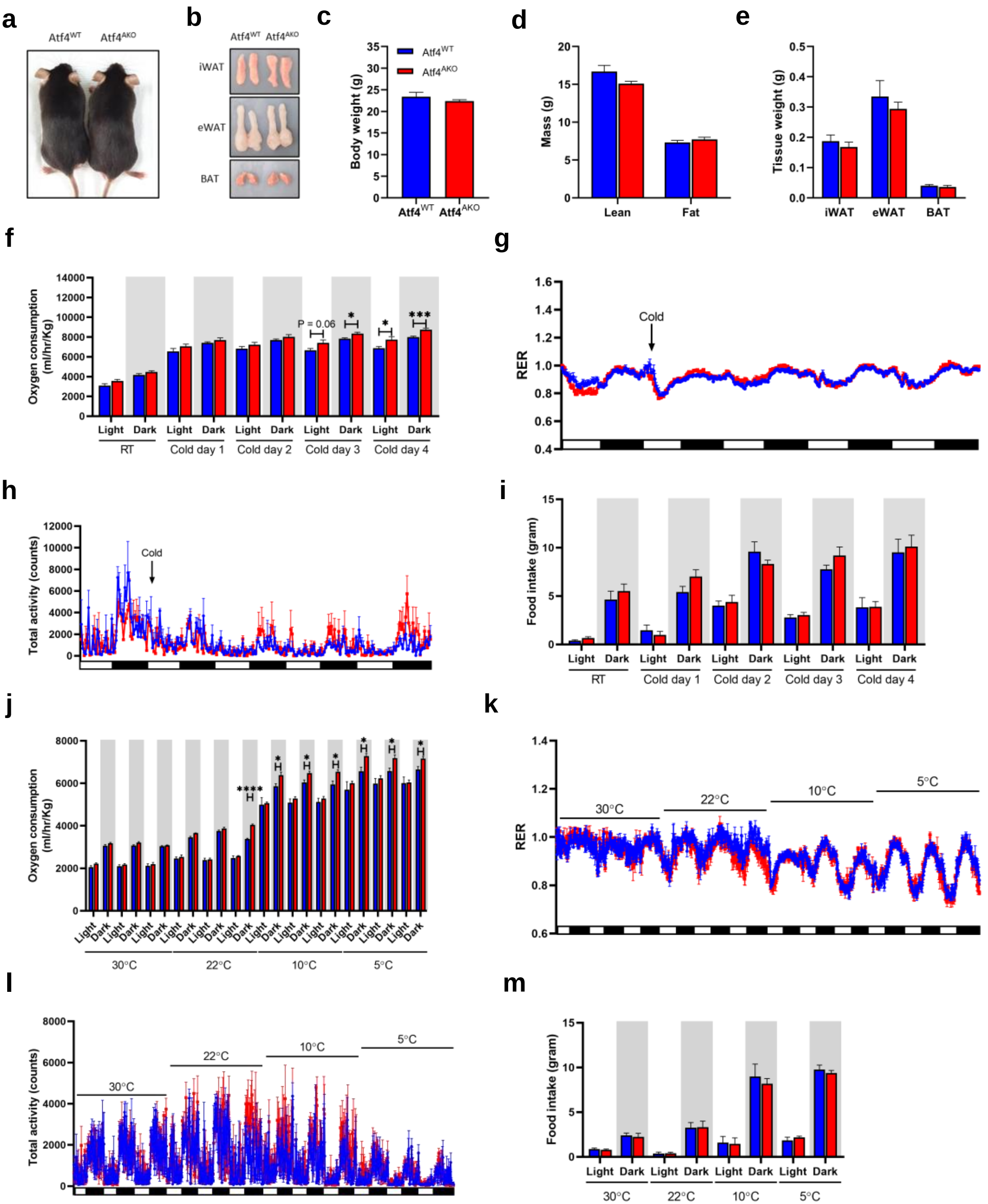

Supplementary Fig. 3

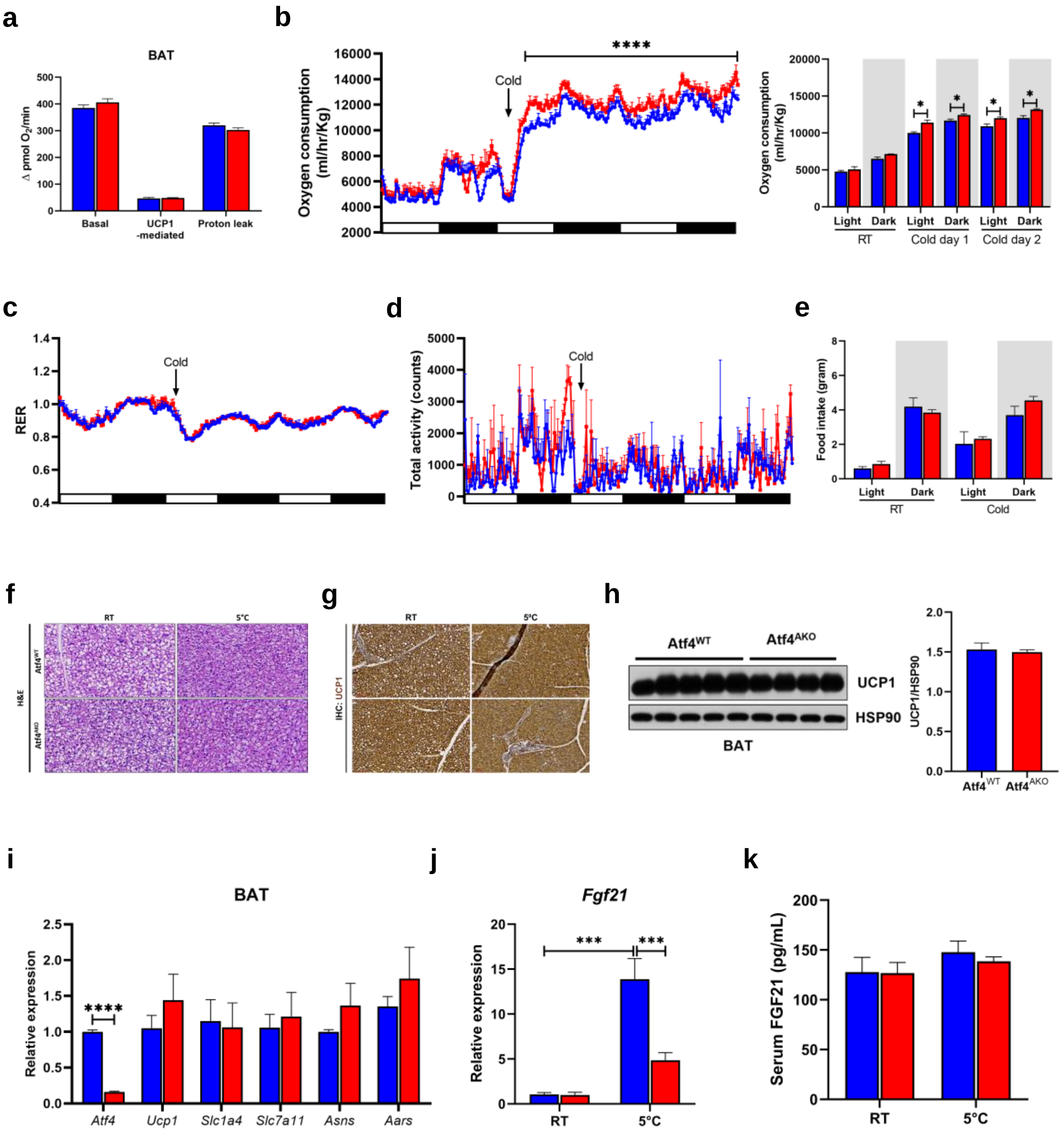

Supplementary Fig. 4

a

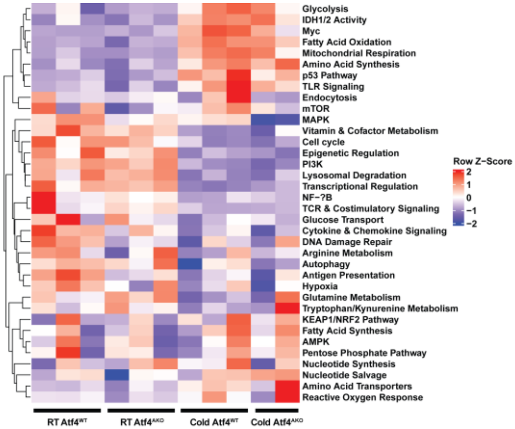

b

Gene	logFC	P-value
Il6	1.324218	0.000602443
Cyp1a1	1.097973	0.028833831
Cacnb4	0.98931	0.006925386
Gapdhs	0.957132	0.012038303
Dao	0.865714	0.00529151
Pnoc	0.7733	0.017787527
Tnf	0.71162	0.015825574
H2-Q1	0.682564	0.011335426
Th	-0.59107	0.021630273
Gtse1	-0.68988	0.047188583
Tymp	-0.81206	0.045574492
Pycr1	-0.82897	0.003458284
Cd180	-0.90714	0.047441406
Cd209e	-0.91334	0.002642426
Tdo2	-0.93155	0.018238397
Fanci	-0.99736	0.043236236
Bub1b	-1.05403	0.039536088
Slc7a5	-1.07498	0.029295619
Pclaf	-1.14052	0.028516268
Upp2	-1.25841	0.001066625
Prodh2	-1.32065	0.013752802
Atf4	-2.40453	0.000000090

Supplementary Fig. 5

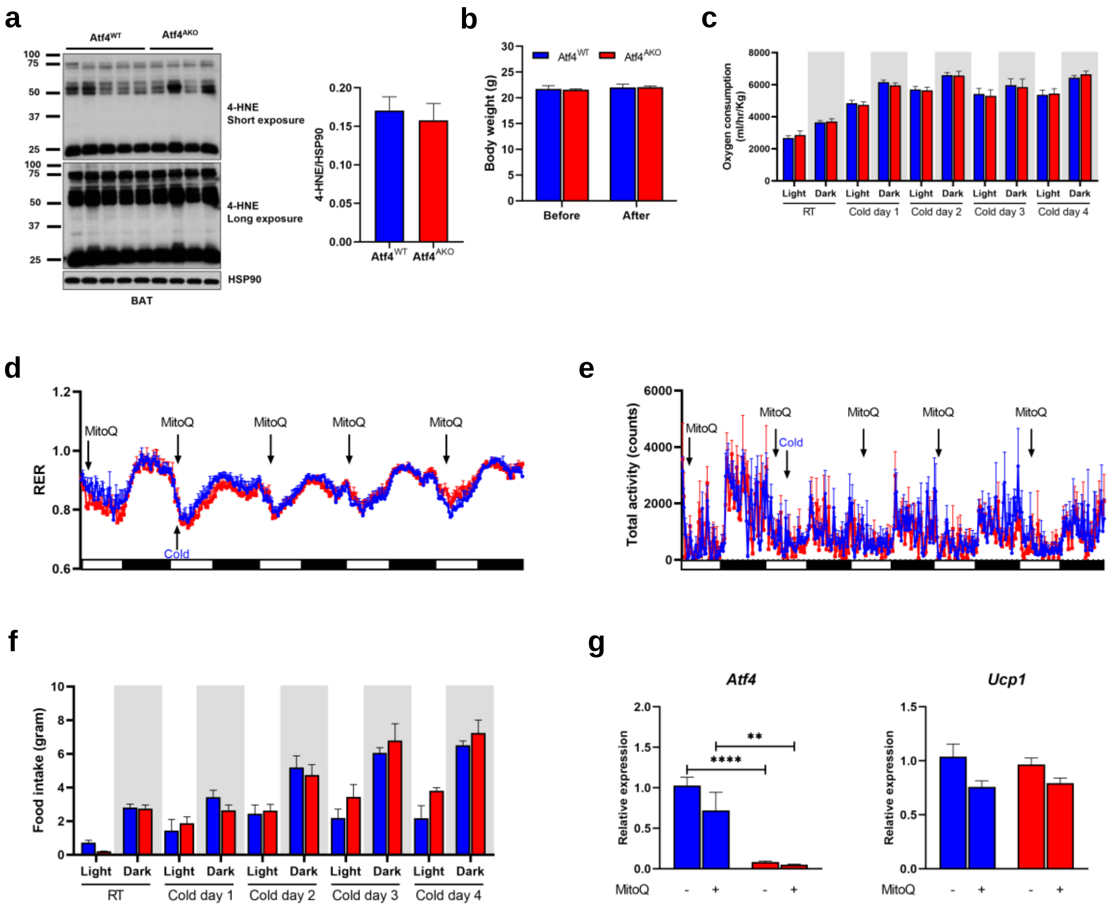

Supplementary Fig. 6

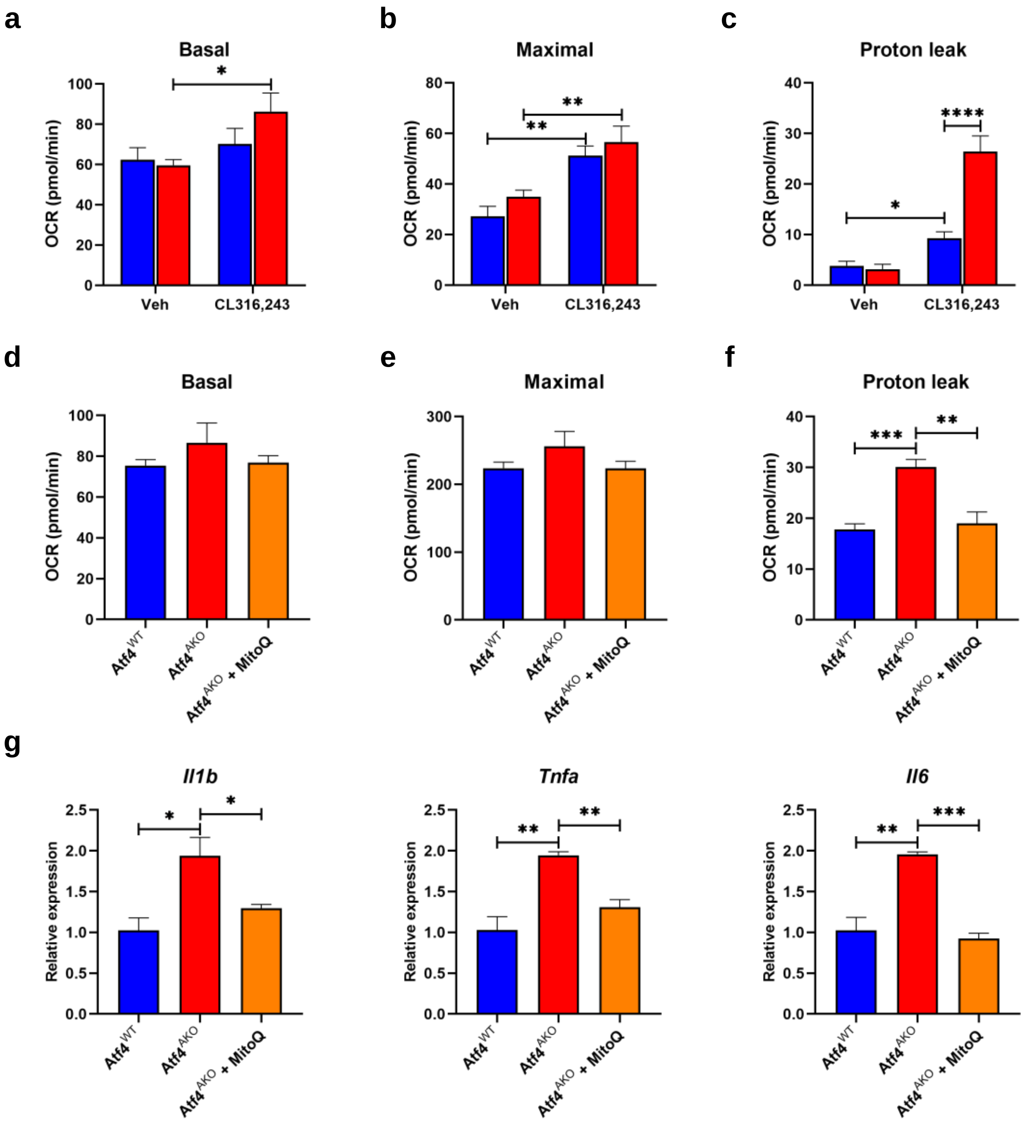

Supplementary Fig. 7

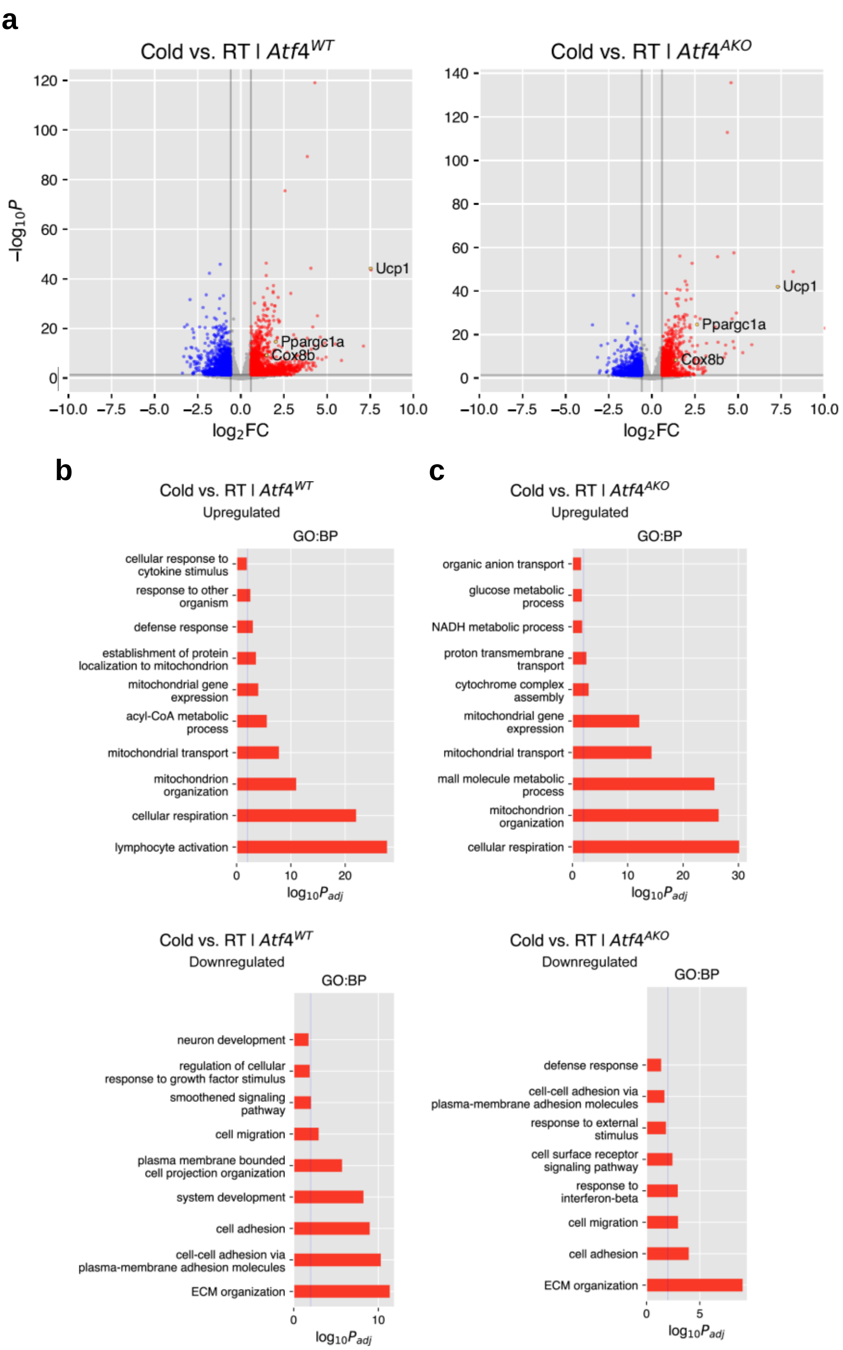

Supplementary Fig. 8

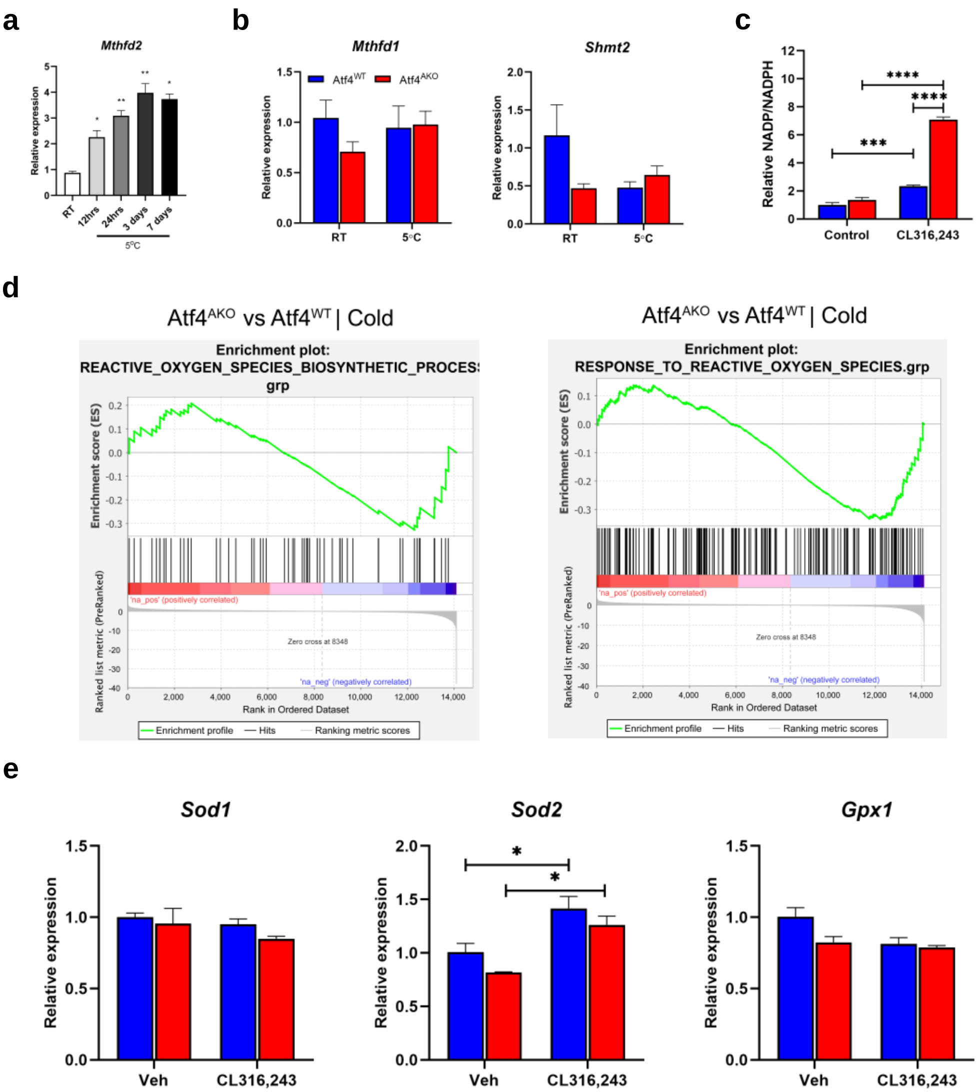

Supplementary Fig. 9

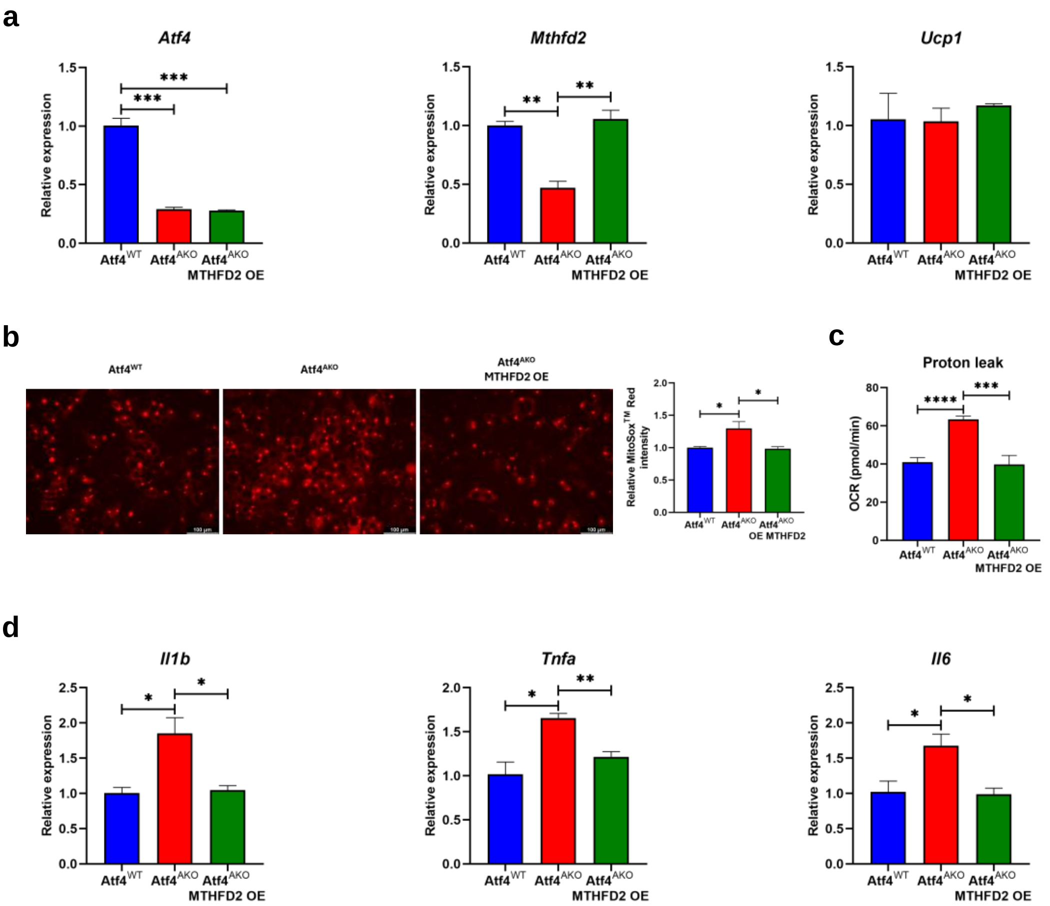

Supplementary Fig. 10

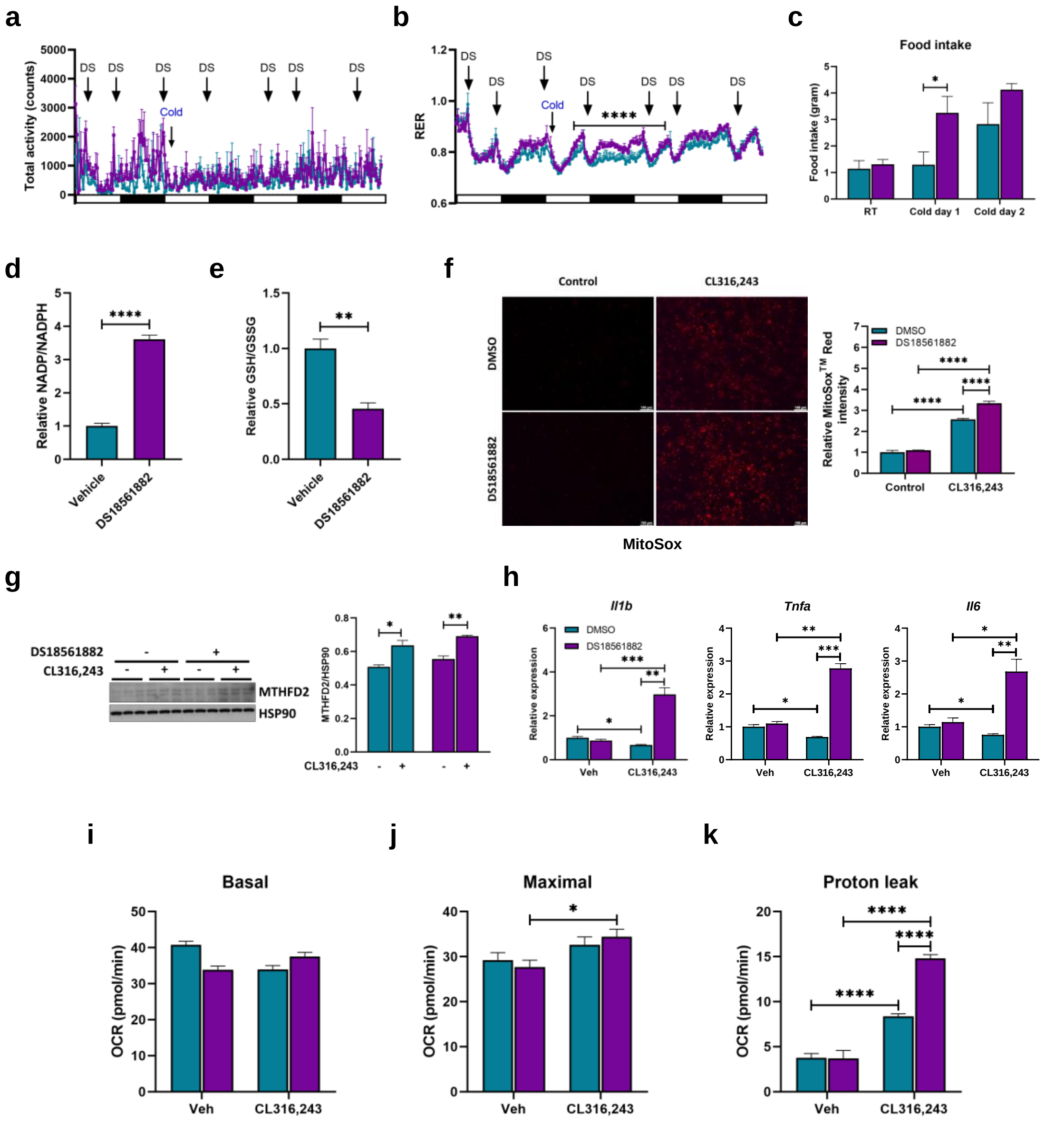

Supplement: Supplementary file 1 — Fig. S1. Thermogenic activation precedes mitochondrial biogenesis during cold-induced iWAT browning. (a-e) Quantitative RT-PCR analysis of thermogenic genes (a) and mitochondria OXPHOS complex genes (b), representative immunoblot analysis for UCP1 and OXPHOS complexes and densitometric quantification of UCP1 and OXPHOS complexes normalized to HSP90 (n = 5 per time point) (c), ratio of mitochondrial to nuclear genomic DNA reflecting mitochondrial number (n = 5 per group) (d), and total mitochondrial protein (n = 4 per group) (e) from iWAT of 10-week-old C57BL/6 male mice subjected to 5 °C for the indicated times. (f) Basal oxygen consumption rate (OCR) and UCP1-mediated OCR of mitochondria isolated from iWAT of 10-week-old C57BL/6 male mice subjected to 5 °C for the indicated times, as measured by using Seahorse XF96 Extracellular Flux analyzer (n = 4 per group). (g–h) Quantitative RT-PCR analysis of mitochondrial-stress-related genes (Hspe1, Hspd1, Hsp70) (g) and ER-stress-related genes (Grp78, Herpud1, and sXbp1/tXbp1) (h) in iWAT from 10-week-old C57BL/6 male mice subjected to 5 °C for the indicated times (n = 5 per group). Data are presented as mean ± SEM. ∗∗∗p < 0.005, ∗∗p < 0.01, ∗p < 0.05. Fig. S2. Real-time metabolic assessment of Atf4WT and Atf4AKO mice. (a–e) Representative images of Atf4WT and Atf4AKO mice (a), iWAT, eWAT, and BAT (b), body weight (c), body composition (d), and tissue weight (e) from Atf4WT and Atf4AKO mice at room temperature (n = 4 for Atf4WT; n = 5 for Atf4AKO). (f–i) Assessment of oxygen consumption (bar graph) (f), RER (g), total activity (h), and food intake (i) in 10-week-old Atf4WT and Atf4AKO mice housed at different ambient temperatures (n = 6 per genotype). (j–m) Assessment of oxygen consumption (bar graph) (j), RER (k), total activity (l), and food intake (m) in 10-week-old Atf4WT and Atf4AKO mice housed at various temperatures (n = 3 per genotype). Data are presented as mean ± SEM. ∗∗∗p < 0.005, ∗∗p < 0.01, ∗p < 0.05. Fig. S3. [file mmc1.pdf]
