# Supplementary material for: Regulation of redox homeostasis by ATF4-MTHFD2 axis during white adipose tissue browning
Source: Redox Biol. 2025 Jun 9;85:103715. doi: 10.1016/j.redox.2025.103715 (PMC12205661; doi:10.1016/j.redox.2025.103715)
Supplement: Multimedia component 2 [file mmc2.docx]

**Table S1. Primer sequences used for RT-PCR analysis in the reported studies**

| 36b4  AAC GGC AGC ATT TAT AAC CC  CGA TCT GAC ACA CAC TG | Itgax  CTG GAT AG CCT TTC TTC TGC TG  GCA CAC TGT GTC CGA ACT C |
| --- | --- |
| Aars  ATG GAT GCC ACT TTA ACA GCA  TGG GTC GAT TGT GTT TAG GAA GA | Mgl1  TGA GAA AGG CTT TAA GAA CTG GG  GAC CAC CTG TAG TGA TGT GGG |
| Arg1  AAC ACG GC AGT GGCT TTAA C  GTC AGT CCC TGG CTT ATG GTT | Mthfd1  GTC GTG CAT CTC TCA CAG AAG  TGA GGG CAG TTG GTT TTA TCT TT |
| Asns  GCA GTG TCT GAG TGC GAT GAA  TCT TAT CGG CTG CAT TCC AAA C | Mthfd2  GTG CTT GGA CCA GTA CTC TAT G  CCT AAG GTT GGA ATG CCT GT |
| Atf4  TGG CCA AGC ACT TGA AAC  AAA GGC ATC CTC CTT GC | Nd1  TCC GAG CAT CTT ATC CAC GC  GTA TGG TGG TAC TCC CGC TG |
| Atp5f1a  TCT CCA TGC CTC TAA CAC TCG  CCA GGT CAA CAG ACG TGT CAG | Nd6  CCC GCA AAC AAA GAT CAC CC  TCT TGA TGG TTT GGG AGA TTG GT |
| Cd206  CAG GTG TGG GCT CAG GTA GT  TGT GGT GAG CTG AAA GGT GA | Nduf8b  TGT TGC CGG GGT CAT ATC CTA  AGC ATC GGG TAG TCG CCA TA |
| Chop  CTG CCT TTC ACC TTG GAG AC  CGT TTC CTG GGG ATG AGA TA | Pgc1a  GCA CCA GAA AAC AGC TCC AAG  CGT CAA ACA CAG CTT GAC AGC |
| Cox4i1  ATG TCA CGA TGC TGT CTG GC  GTG CCC CCG TTC ATC TCG GC | Prdm16  CAG CAC GGT GAA GCC ATT C  GCG TGC ATC CGC TTG TG |
| Cox8b  TGC TGG AAC CAT GAA GCC AAC  AGC CAG CCA AAA CTC CCA CTT | Sdhb  AAT TTG CCA TTT ACC GAT GGG A  AGC ATC CAA CAC CAT AGG TCC |
| Cytb  TGT ATA CGC CAT TCT ACG CT  AGG CTT CGT TGC TTT GAG GT | Shmt2  TGG CAA GAG ATA CTA CGG AGG  GCA GGT CCA ACC CCA TGAT |
| Fabp4  TCA CGC CTT TCA TAA CAC ATT CC  AAG GTG AAG AGC ATC ATA ACC CT | Slc1a4  GGC ATC GCT GTT GCT TAC TTC  CGA GGA AAG AGT CCA CTG TCT |
| Fgf21  CTC TAT GGA TCG CCT CAC TTT G  GGT ACA CAT TGT AAC CGT CCT C | Slc7a11  CTG GGA CTT CCG TGG ACC T  TCT TGC AGA CGA TAC GCA GAA |
| Gpx1  AGT CCA CCG TGT ATG CCT TCT  GAG ACG CGA CAT TCT CAA TGA | Sod1  AAC CAG TTG TGT TGT CAG GAC  CCA CCA TGT TTC TTA GAG TGA GG |
| Grp78  GGT GCA GCA GGA CAT CAA GTT  CCC ACC TCC AAT ATC AAC TTG A | Sod2  CAG ACC TGC CTT ACG ACT ATG G  CTC GGT GGC GTT GAG ATT GTT |
| Herpud1  CAA CAG CAG CTT CCC AGA AT  CCG CAG TTG GAG TGT GAG T | s-Xbp1  GAG TCC GCA GCA GGT G  GTG TCA GAG TCC ATG GGA |
| Hsp70  CAA GCT GTC ACC AAT CCA AAC  CAA CCC AAG CAT CAC CAT TG | t-Xbp1  AAG AAC ACG CTT GGG AAT GG  ACT CCC CTT GGC CTC CAC |
| Hspd1  AGT GTT CAG TCC ATT GTC CC  TGA CTG CCA CAA CCT GAA G | Tnfa  CAG GCG GTG CCT ATG TCT C  CGA TCA CCC CGA AGT TCA GTA G |
| Hspe1  GCG AAG GCG AGA GTC ATG  TGC TTG CAA CAC TTT TCC TTG | Ucp1  ACT GCC ACA CCT CCA GTC ATT  CTT TGC CTC ACT CAG GAT TGG |
| Il6  TAG TCC TTC CTA CCC CAA TTT CC  TTG GTC CTT AGC CAC TCC TTC | Uqcrc2  AAA GTT GCC CCG AAG GTT AAA  GAG CAT AGT TTT CCA GAG AAG CA |
| Il1b  GCA ACT GTT CCT GAA CTC AAC T  ATC TTT TGG GGT CCG TCA ACT |  |
| iNos  CCA AGC CCT CAC CTA CTT CC  CTC TGA GGG CTG ACA CAA GG |  |

**Table S2. List of reagent and resources used in the reported studies**

| **REAGENT or RESOURCE** | **SOURCE** | **IDENTIFIER** |
| --- | --- | --- |
| **Antibodies** | | |
| Anti-UCP1 | Abcam | Cat#ab10983; AB_2241462 |
| Anti-eIF2α | Thermo Fisher | Cat#AHO1182; AB_2536326 |
| Anti-P- eIF2α | Abcam | Cat#ab32157; AB_732117 |
| Anti-Total OXPHOS | Abcam | Cat#ab110413; AB_2629281 |
| Anti-4-HNE | Invitrogen | Cat#MA5-27570; AB_2735095 |
| Anti-ATF4 | Cell Signaling | Cat#11815; AB_2616025 |
| Anti-HSP90 | Santa Cruz | Cat#sc-13119; AB_675659 |
| Anti-Rabbit-HRP | Cell Signaling | Cat#7074; AB_2099233 |
| Anti-Mouse-HRP | Cell Signaling | Cat#7076; AB_330924 |
| Anti-CD45 | Thermo Fisher | Cat#48-0451-82; AB_1518806 |
| Anti-F4/80 | Biolegend | Cat#123114; AB_893478 |
| Anti-CD11b | Thermo Fisher | Cat#11-0112-82; AB_464935 |
| Anti-CD206 | BD Biosciences | Cat#565250; AB_2739133 |
| Anti-CD11c | Thermo Fisher | Cat#12-0114-83; AB_465553 |
| Live/dead | Thermo Fisher | Cat#65-0866-14 |
| **Chemicals, peptides, and recombinant proteins** | | |
| Collagenase Type 1 | Worthington | Cat#LS004196 |
| FBS | Corning | Cat#35-015-CV |
| DMEM | Hyclone | Cat#SH30243.01 |
| Trypsin | Corning | Cat#25-053-CI |
| Trizol Ambion | Invitrogen | Cat#15596018 |
| MitoQ | MedChemExpress | Cat#845959-50-4 |
| CL316,243 | Sigma | Cat#C5976 |
| DS18561882 | MedChemExpress | Cat#HY-130251 |
| Oligomycin | Sigma | Cat#75351 |
| FCCP | Sigma | Cat#C2920 |
| Rotenone | Sigma | Cat#R8875 |
| Antimycin A | Sigma | Cat#A8674 |
| Lenti-X concentrator | TaKaRa | Cat#631232 |
| EZ-Fusion™ HT Cloning core Kit | Enzynomic | Cat#EZ016TM |
| **Critical commercial assays** | | |
| MitoSox^TM^ Red | Invitrogen | Cat#M36008 |
| DCFDA/H2DCFDA | Abcam | Cat#ab113851 |
| NADP/NADPH Assay | Abcam | Cat#ab65349 |
| GSH/GSSG Ratio Assay | Abcam | Cat#ab138881 |
| Mitochondria Isolation Kit | Abcam | Cat#ab65320 |
| Mouse and Rabbit Specific HRP/DAB Detection IHC Kit | Abcam | Cat#ab64264 |
| SYBR Green Realtime PCR Master Mix | Toyobo | Cat#QPK-201 |
| ReverTra Ace^TM^ qPCR RT Master Mix | Toyobo | Cat#FSQ-201 |
| **Experimental models: Organisms/strains** | | |
| C57BL/6N |  |  |
| B6.FVB-Tg(Adipoq-Cre)1Evdr/J | Jackson Laboratory | Cat#028020; IMSR_JAX:028020 |
| **Software** | | |
| Prism 8 | Graphpad | RRID: SCR_000306 |
| ImageJ | <http://imagej.nih.gov> | RRID: SCR_003070 |
| Flowjo v.10.8.1 | Tree Star | RRID: SCR_008520 |
| R | Lucent Technologies | RRID: SCR_001905 |
| nSolver™ v.4.0 | NanoString | RRID: SCR_003420 |
| BioRender | BioRender Software | RRID: SCR_018361 |

**RESOURCE AVAILABILITY**

**Lead contact**

Further information and requests for reagents and resources should be directed to and will be fulfilled by the lead contacts, Min-Woo Lee ([mwlee12@sch.ac.kr](mailto:mwlee12@sch.ac.kr)).

**Data and code availability**

- Data: All data reported in this paper will be shared by the lead contact upon request.
- Code: This paper does not report original code.
- Any additional information required to reanalyze the data reported in this paper is available from the lead contact upon request.
